# Comprehensive Phenotyping of Circulating T- and B-Cell Subsets in Patients with Ankylosing Spondylitis Associated with Crohn’s Disease

**DOI:** 10.3390/cells15171580

**Published:** 2026-08-31

**Authors:** Artem Rubinstein, Arthur Aquino, Denis Davydov, Oksana Shchukina, Zoia Korobova, Jennet Mammedova, Eleonora Starikova, Valerii Marchenko, Michael Galagudza, Igor Kudryavtsev

**Affiliations:** 1 Institute of Experimental Medicine, 197376 St. Petersburg, Russia; jennet_m@mail.ru (J.M.); starickova@yandex.ru (E.S.); igorek1981@yandex.ru (I.K.); 2 Almazov National Medical Research Center, 197341 St. Petersburg, Russia; akino97@bk.ru (A.A.); galagoudza@mail.ru (M.G.); 3 Medical Faculty, First Saint Petersburg State I. Pavlov Medical University, 197022 St. Petersburg, Russia; davydov.rheum@gmail.com (D.D.); burmao@gmail.com (O.S.); marchvn@mail.ru (V.M.); 4 Saint Petersburg Pasteur Institute, 197101 St. Petersburg, Russia; zoia-korobova@yandex.ru; 5 Faculty of Biology, Saint Petersburg State University, 199034 St. Petersburg, Russia

**Keywords:** ankylosing spondylitis, Crohn’s disease, inflammatory bowel disease, Treg, CD73, CD39, CD4+ T cells, CD8+ T cells, follicular T-cell subsets, B cells

## Abstract

**Highlights:**

**What are the main findings?**

- Patients with ankylosing spondylitis associated with Crohn’s disease exhibit decreased effector memory Th and Tcyt cell subsets compared to patients with Crohn’s disease, as well as an increase in T-regulatory cells (Tregs) in the peripheral blood compared with other groups. Phenotypic alterations in the CD39/CD73 profile of Tregs were also observed.
- Patients with ankylosing spondylitis associated with Crohn’s disease exhibit decreased Tfh17 levels compared to patients with ankylosing spondylitis alone, and a decrease in ‘switched’ memory B cells compared to patients with Crohn’s disease alone. Both of these populations were negatively correlated with acute-phase markers.

**What are the implications of the main findings?**

- The observed phenotypic alterations in T cell-mediated adaptive immunity may reflect a predominantly pro-inflammatory profile, with possible signs of diminished regulatory activity.
- The changes noted in the humoral component of the adaptive immune response in this condition might be compensatory in nature and could potentially be associated with resolution of inflammation. However, further functional studies are needed to confirm this hypothesis.

**Abstract:**

**Background:** Ankylosing spondylitis (AS) associated with Crohn’s disease (CD) is a nosological form of spondyloarthropathies with a low population prevalence. The pathogenesis of this disease is not fully understood, and there are no precise differential diagnostic methods to distinguish AS associated with CD from AS and CD separately in the early stages of the disease. The main objective for this study is to define lymphocyte-mediated immunity in patients with AS associated with CD. **Methods:** For the pilot study, we recruited four groups: CD (n = 16), AS+CD (n = 13), AS (n = 13) and healthy controls (HC, n = 26). Immune phenotyping of peripheral blood lymphocytes was carried out via flow cytometry. **Results:** In the AS+CD group, circulating regulatory T-cell (Treg) levels were increased compared to the other groups. The frequency of CD73+ Tregs within the effector memory (EM) compartment was also elevated in the AS+CD group relative to the AS and CD groups alone. At the same time, the EM Tcyt and EM Th populations were lower in the AS+CD group compared to the CD group. Tfh17 levels were lower in the AS+CD group than in the AS group and showed a positive correlation with Bm5 and ‘switched’ memory B cells. Additionally, Tfh17 and ‘switched’ memory B cells correlated negatively with acute-phase markers, whereas Tfh2 correlated positively with the BASDAI activity index. **Conclusions:** In patients with AS associated with CD, exhaustion of the regulatory compartment of adaptive immunity is seen, whereas the humoral component appears oriented towards resolving the inflammatory responses.

## 1. Introduction

Ankylosing spondylitis (AS) is a chronic inflammatory disease within the spondyloarthritis (SpA) group, characterized by involvement of the spine, peripheral joints, and entheses. Extraskeletal structures, including the gastrointestinal tract (GIT), may also be affected. Crohn’s disease (CD), conversely, is an inflammatory bowel disease (IBD) that, beyond gastrointestinal manifestations, can involve extraintestinal sites such as the axial skeleton and/or peripheral joints [1,2]. IBD-associated SpA is a subtype of spondyloarthritis occurring in patients with inflammatory bowel disease. This nosological form is also included in the SpA classification proposed by the Assessment of SpondyloArthritis international Society (ASAS) [3]. Up to 50% of patients with IBD have musculoskeletal manifestations. AS is detected in 16% of patients, sacroiliitis at various stages is found in 12–20% of cases, and peripheral arthritis affects 12–20% of patients with IBD [4]. It should be noted that the epidemiology of SpA associated with IBD depends on the characteristics of the diagnostic criteria used, as well as the year of their introduction into clinical practice and subsequent updates over time [2]. A recent 20-year study of a Norwegian IBD cohort (441 patients) showed that 36% of patients met the ASAS criteria for SpA, of which 8% met the criteria for axial SpA (axSpA) and 28% for peripheral SpA [5]. In a study of 50 patients with CD presenting with inflammatory back pain, 28% of them met AS according to the modified New York criteria [6]. In patients with IBD, an asymptomatic course of inflammatory arthropathy is often observed. For instance, in the study by Scott et al., radiography and computed tomography (CT) of the sacroiliac joints (SIJ) were performed in patients with CD to detect changes consistent with sacroiliitis. As a result, sacroiliitis was found in 29% of patients with CD, while symptomatic disease was observed in only 3% of cases [7]. Conversely, SpA patients have a high risk of IBD in the first year of disease manifestation [8]. Recent study showed prevalence of IBD of 14% in AS patients [9]. Notably, data indicate subclinical gastrointestinal involvement in patients with SpA. Accordingly, up to 44% of SpA patients present with intestinal lesions that are detectable only via endoscopy [10]. Furthermore, microscopic inflammation of the intestinal wall is observed in 70% of patients with SpA [11]. The cumulative prevalence of IBD in combination with SpA is a matter of the discussion. Stolwijk et al. demonstrated an increase in the incidence of IBD from 3.4% at the time of diagnosis of SpA to 7.5% 20 years after diagnosis verification [8]. Another meta-analysis, however, did not demonstrate any link between disease duration and IBD prevalence [12].

Due to similar prevalence of IBD in SpA patients and SpA in IBD patients, different experts study whether these two groups are also similar in terms of the disease development. Given the efficacy of target anti-IL-23/IL-17A therapy, it is reasonable to assume that type III immune responses are involved in both conditions. The question of where the primary immune response originates, however, remains open, though a modern concept exists suggesting involvement of the intestinal mucosa [13]. There is also evidence of differences in the pathogenesis of diseases belonging to the IBD and SpA groups. For example, in CD, the cytokine IL-17A is produced in an IL-23-dependent manner, whereas in AS, it is IL-23 independent. Furthermore, it has been suggested that the main producers of IL-17A in AS are innate immune cells (ILC3s and γδT lymphocytes), while in CD, they are adaptive immune cells (CD4+ and CD8+ T lymphocytes) [14]. Phenotypes of immune cells playing a key role in SpA associated with IBD are not fully defined at this point.

The main objective of this study was to identify phenotypic features of peripheral blood lymphocytes in patients with AS associated with CD, as well as to detect differences between the phenotypic characteristics of lymphocytes in these patients and those in patients with AS alone (without gastrointestinal involvement) and CD alone (without musculoskeletal involvement).

## 2. Materials and Methods

### 2.1. Patient Cohorts

This study included 13 patients with AS associated with CD (group AS+CD), 16 patients with CD (group CD), and 26 healthy controls (group HC). All participants were similarly distributed based on age and sex. AS diagnosis was verified based on Modified New York Criteria of 1984. Active disease, defined by the validated activity indices BASDAI (Bath Ankylosing Spondylitis Disease Activity Index) and ASDAS (Ankylosing Spondylitis Disease Activity Score) based on peripheral blood C-reactive protein levels, was required for inclusion. CD diagnosis was established based on gastroenterologist exam at a specialized IBD center based on clinical, laboratory, endoscopic, and morphological data. Active CD, defined by the Best’s Crohn’s Disease Activity Index (CDAI) and Harvey–Bradshaw index, was required for inclusion. The diagnosis of AS associated with CD was confirmed in cases where patients simultaneously met the diagnostic criteria for both conditions (groups AS and CD). We excluded patients with oncology history, pregnancy, other rheumatic disease, chronic diseases in acute phase, acute and chronic infectious diseases, and high doses of glucocorticoids intake. Detailed clinical information is presented in Table 1. The study object was peripheral blood with K3EDTA in a 4 mL tube. Immune phenotyping was performed within 2–4 h after blood collection. Staining for B-cell, T-cell, and Treg populations was performed in three separate tubes. The markers used and their corresponding fluorochrome conjugates are presented below.

### 2.2. B Cell Immunophenotyping by Flow Cytometry

The frequency and phenotype of B-cell subsets in the peripheral blood samples from three groups of patients with CD, CD+AS, and AS were analyzed by flow cytometry as it was described previously [15]. In brief, 100 μL of whole peripheral blood was stained (15 min in dark at room temperature) with Alexa Fluor 488-labelled mouse anti-human IgD (clone IA6-2, cat. 348216, BioLegend, San Diego, CA, USA), PE-labelled mouse anti-human CD38 (clone LS198-4-3, cat. A07779, Beckman Coulter, Brea, CA, USA), PC7-labelled mouse anti-human CD27 (clone 1A4CD27, cat. A54823, Beckman Coulter, Brea, CA, USA), APC/Cy7-labelled CD19 (clone HIB19, cat. 302218, BioLegend, San Diego, CA, USA), Pacific Blue-labelled mouse anti-human CD5 (clone BL1a, cat. A82790, Beckman Coulter, Brea, CA, USA), and Krome Orange-labelled mouse anti-human CD45 (clone J33, cat. A96416, Beckman Coulter, Brea, CA, USA). The staining protocols were performed according to the manufacturer’s recommendations. Afterward, 1000 µL of Versa-Fix solution (975 µL of VersaLyse Lysing Solution (Beckman Coulter, Brea, CA, USA) supplied with 25 μL of IOTest 3 Fixative Solution (Beckman Coulter, Brea, CA, USA)) was introduced into blood samples, followed by gentle mixing. It was subsequently incubated for a further 15 min at room temperature in dark. Next, all blood samples were washed (7 min, 330 g) twice with 4.5 mL of wash buffer (sterile phosphate-buffered saline (PBS) containing 2% of heat inactivated fetal bovine serum, Sigma-Aldrich, St. Louis, MO, USA)), and resuspended in 500 μL of fresh PBS with 2% neutral formalin (cat. HT5011-1CS, Sigma-Aldrich, St. Louis, MO, USA), and subjected to flow cytometry analysis. At least 5000 CD19+ B cells were analyzed for each sample. The gating strategy and algorithms used for B-cell subset analysis were described previously in details [15], and presented in Supplementary Materials (Figure S1, Supplementary Table S1).

### 2.3. T-Cell Immunophenotyping by Flow Cytometry

CD4+ and CD8+ T-cell subsets were analyzed using multicolor flow cytometry [16]. Peripheral blood (100 μL) samples were stained using FITC-labelled mouse anti-human CD45RA (clone ALB11, cat. IM0584U, Beckman Coulter, Brea, CA, USA), PE-labelled mouse anti-human CD62L (clone DREG56, cat. IM2214U, Beckman Coulter, Brea, CA, USA), PerCP/Cy5.5-labelled mouse anti-human CXCR5 (CD185, clone J252D4, cat. 356910, BioLegend, San Diego, CA, USA), PE/Cy7-labelled mouse anti-human CCR6 (CD196, clone G034E3, cat 353418, BioLegend, San Diego, CA, USA), APC-labelled mouse anti-human CXCR3 (CD183, clone G025H7, cat. 353708, BioLegend, San Diego, CA, USA), APC-Alexa Fluor 750-labelled mouse anti-human CD3 (clone UCHT1, cat. A94680, Beckman Coulter, Brea, CA, USA), Pacific Blue-labelled mouse anti-human CD4 (clone 13B8.2, cat. B49197, Beckman Coulter, Brea, CA, USA), and Brilliant Violet 510-labelled mouse anti-human CCR4 (CD194, clone L291H4, cat. 359416, BioLegend, San Diego, CA, USA). The staining protocols were performed according to the manufacturer’s recommendations. In brief, 100 μL of whole peripheral blood was stained with the above-mentioned antibodies at room temperature for 15 min in the dark. After antibody staining, erythrocytes were lysed by adding 925 µL of VersaLyse Lysing Solution (Beckman Coulter, Brea, CA, USA) supplied with 25 μL of IOTest 3 Fixative Solution (Beckman Coulter, Brea, CA, USA) in the dark at room temperature for 15 min. Finally, all samples were washed with 4.5 mL of wash buffer (sterile phosphate-buffered saline (PBS) containing 2% of heat inactivated fetal bovine serum, Sigma-Aldrich, St. Louis, MO, USA)), and resuspended in 500 μL of fresh PBS with 2% neutral formalin (cat. HT5011-1CS, Sigma-Aldrich, St. Louis, MO, USA), and subjected to flow cytometry analysis. At least 40,000 CD3+CD4+ Th cells were collected from each sample. The gating strategy is presented in Supplementary File (Figure S2, Supplementary Table S2).

### 2.4. Regulatory T-Cell Immunophenotyping by Flow Cytometry

Regulatory T cells (Treg) and their main subsets were analyzed using multicolor flow cytometry as it was described previously in details [17]. In brief, 100 μL of peripheral blood was stained in with the following combination of mouse anti-human antibodies: CD39-FITC (clone A1, cat. 328206, BioLegend, San Diego, CA, USA), CD25-PE (clone B1.49.9, cat. A07774, Beckman Coulter, Brea, CA, USA), CD62L-ECD (clone DREG56, cat. IM2713U, Beckman Coulter, Brea, CA, USA), CD45RA-PerCP/Cyanine5.5 (clone HI100, cat. 304122, BioLegend, San Diego, CA, USA), CD4-PC7 (clone SFCI12T4D11 (T4), cat 737660, Beckman Coulter, Brea, CA, USA), CD8-APC (clone B9.11, cat. IM2469, Beckman Coulter, Brea, CA, USA), CD3-APC-Alexa Fluor 750 (clone UCHT1, cat. A94680, Beckman Coulter, Brea, CA, USA), CD73-Pacific Blue (clone AD2, cat. 344012, BioLegend, San Diego, CA, USA), and CD45-Krome Orange (clone J33, cat. A96416, Beckman Coulter, Brea, CA, USA). Staining protocols were performed in accordance with the manufacturer’s recommendations. All samples were stained with antibodies in the dark at room temperature for 15 min. Next, red blood cells in the blood samples were lysed by adding 975 μL VersaLyse Lysing Solution supplied with 25 μL of IOTest 3 Fixative Solution (cat. A09777 and cat. A07800, Beckman Coulter, Brea, CA, USA) in the dark at room temperature for 15 min. Next, all samples were washed once with PBS for 7 min at 330 g. After being washed with PBS, the cells were re-suspended in 250 μL of fresh PBS supplied with 2% of neutral formalin (cat. HT5011-1CS, Sigma-Aldrich, St. Louis, MO, USA), and were subjected to flow cytometry analysis. At least 10,000 of CD3+CD4+CD25bright Treg cells were collected from each sample. The gating strategy and algorithms used for Treg cell subset analysis were described previously in details [17], and presented in Supplementary File (Figure S3, Supplementary Table S3).

### 2.5. Statistical Analysis

Flow cytometry data analysis was performed with FlowJo™ v10 Software (BD Bioscience Inc., San Jose, CA, USA), and Kaluza™ software v2.3 (Beckman Coulter, USA).

Statistical analysis was performed using GraphPad Prism 8 (GraphPad Software, San Diego, CA, USA) and Statistica 7.0 (StatSoft, Tulsa, OK, USA). The results are presented as the percentage of the parent population, expressed as median and interquartile range—Me (Q25; Q75). Normality was assessed using the Shapiro–Wilk and Kolmogorov–Smirnov goodness-of-fit tests. According to these tests, the distribution of lymphocyte subpopulation data in all patient groups and healthy controls deviated significantly from normality (*p* < 0.05), confirming their non-parametric nature. Pairwise comparisons of non-parametric independent samples were performed using the Mann–Whitney U test. Differences were considered statistically significant at *p* < 0.05.

## 3. Results

### 3.1. Alterations in Major Peripheral Blood Lymphocyte Populations in Patient with CD, AS+CD, and AS

**Figure 1:**
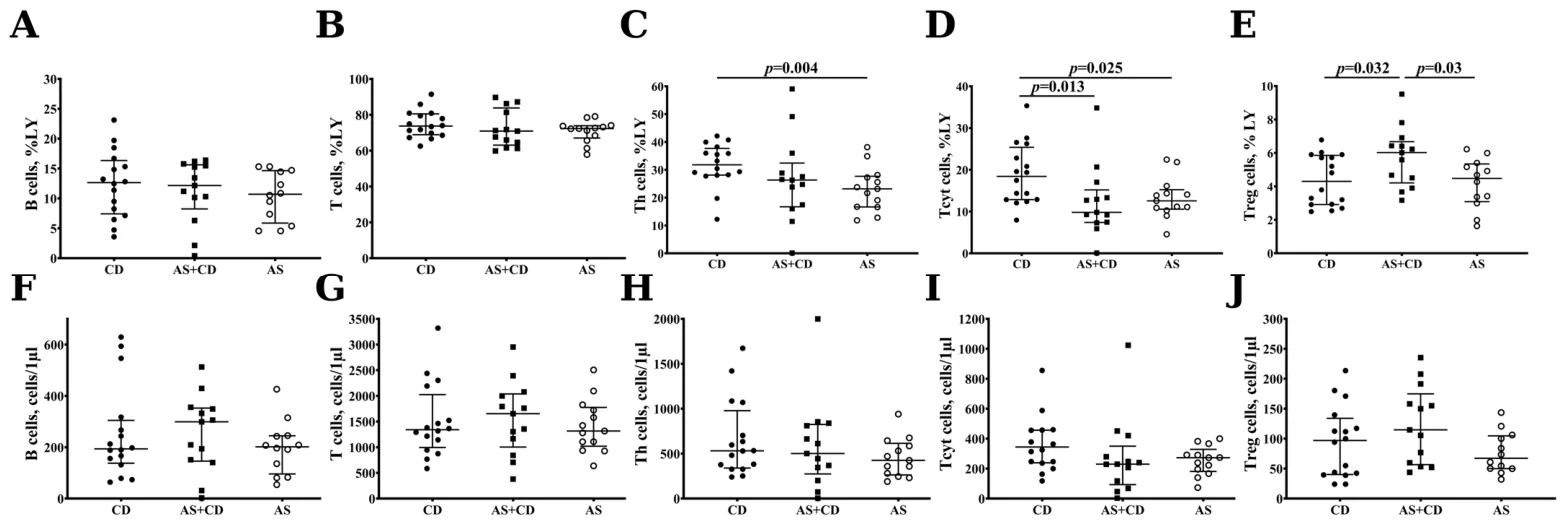
Comparison of relative and absolute frequencies of major lymphocyte populations in patients with CD, AS+CD, and AS. Scatter plots (**A**–**J**) show percentages and absolute counts of B cells, T cells, Th cells, Tcyt cells, and Tregs. Black circles represent patients with Crohn’s disease (CD, n = 16); black squares represent patients with AS associated with CD (AS+CD, n = 13); open circles represent patients with ankylosing spondylitis alone (AS, n = 13). Each symbol corresponds to an individual subject. Horizontal lines indicate the median and interquartile range (Med (Q25; Q75)). Statistical comparisons were performed using the Mann–Whitney U test.

To compare absolute and relative levels of major lymphocyte populations, we analyzed two lineage surface markers—CD3 and CD19. To characterize cytotoxic T cells (Tcyt), T-helper cells (Th), and regulatory T cells (Treg), we measured CD4, CD8, and CD25 expression. This approach allowed us to distinguish the following populations: T cells (CD45+++CD3+), B cells (CD45+++CD19+), Tcyt (CD45+++CD3+CD8+), Th (CD45+++CD3+CD4+), and Treg (CD45+++CD3+CD4+CD25bright) (Figure 1). 

No significant differences in absolute or relative T-cell and B-cell counts were observed among the three groups. The AS+CD group showed elevated Treg percentages compared with the CD and AS groups (6.02% (4.21; 6.67) vs. 4.3% (2.92; 5.86) and 4.48% (3.09; 5.34), respectively; *p* = 0.032 and *p* = 0.030). Absolute Treg counts did not differ between groups.

Circulating Th frequencies were higher in the CD group than in the AS group (31.80% (28.08; 37.68) vs. 23.14% (16.66; 27.68); *p* = 0.004). Tcyt percentages were lower in the AS+CD and AS groups compared with the CD group (9.81% (7.39; 15.19) and 12.56% (10.57; 25.25) vs. 18.42% (12.86; 25.38); *p* = 0.013 and *p* = 0.025, respectively). Absolute Th and Tcyt counts did not differ significantly between the three group.

### 3.2. Imbalance of Memory Subpopulations of Th and Tcyt Cells in Patients with AS+CD

**Figure 2:**
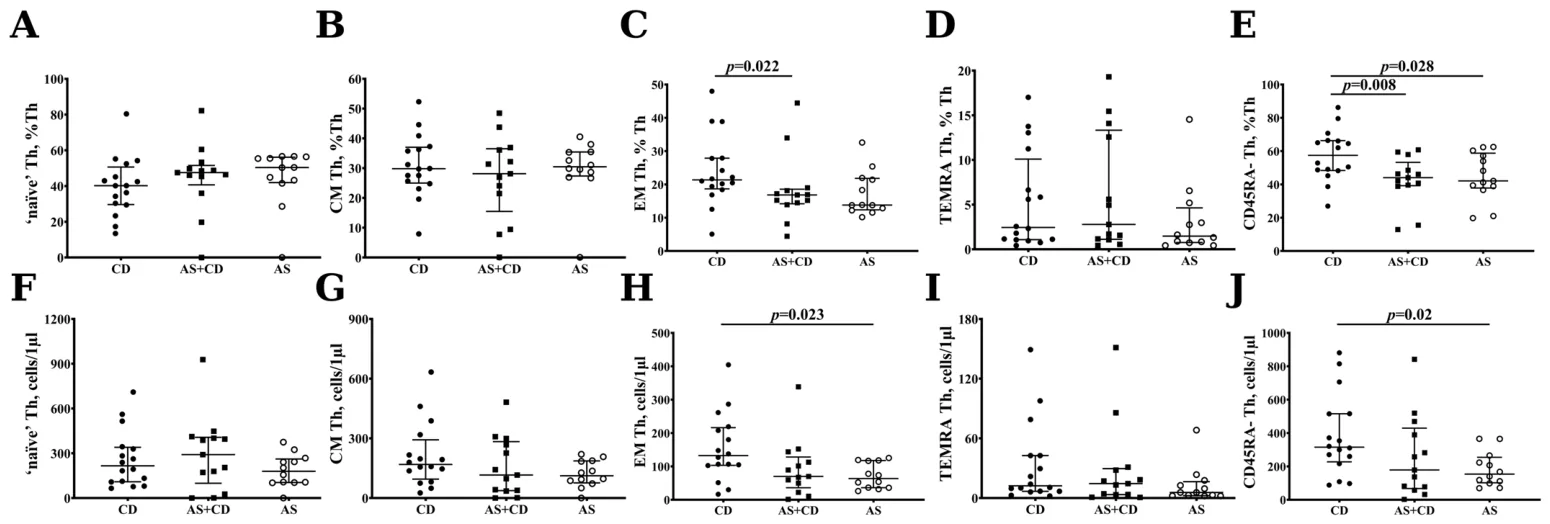
Alterations in Th maturation subsets in patients with CD, AS+CD, and AS. Scatter plots (**A**–**J**) show the percentages and absolute counts of ‘naïve’, central memory (CM), effector memory (EM), effector memory CD45RA-re-expressing (TEMRA) Th cells, and total memory Th cells. Black circles represent patients with Crohn’s disease (CD, n = 16); black squares represent patients with AS associated with CD (AS+CD, n = 13); open circles represent patients with ankylosing spondylitis alone (AS, n = 13). Each symbol corresponds to an individual subject. Horizontal lines indicate the median and interquartile range (Med (Q25; Q75)). Statistical comparisons were performed using the Mann–Whitney U test.

**Figure 3:**
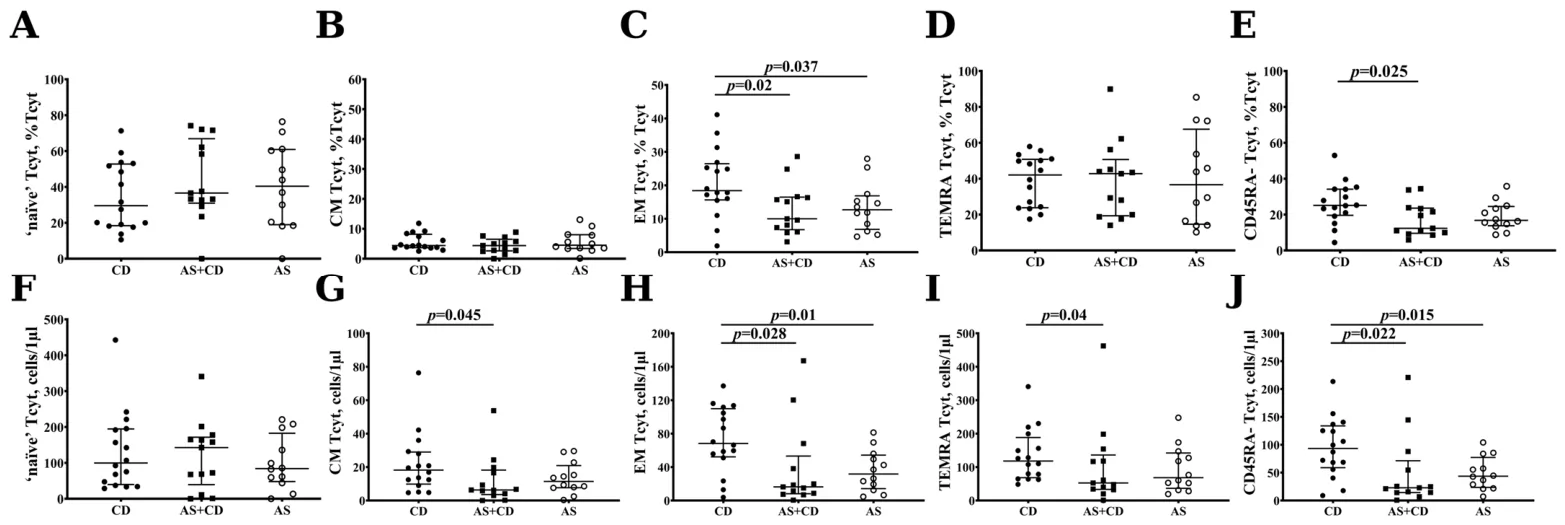
Alterations in Tcyt maturation subsets in patients with CD, AS+CD, and AS. Scatter plots (**A**–**J**) show the percentages and absolute counts of ‘naïve’, central memory (CM), effector memory (EM), effector memory CD45RA-re-expressing (TEMRA) Tcyt cells, and total memory Tcyt cells. Black circles represent patients with Crohn’s disease (CD, n = 16); black squares represent patients with AS associated with CD (AS+CD, n = 13); open circles represent patients with ankylosing spondylitis alone (AS, n = 13). Each symbol corresponds to an individual subject. Horizontal lines indicate the median and interquartile range (Med (Q25; Q75)). Statistical comparisons were performed using the Mann–Whitney U test.

To detect memory T cells, we used phenotyping based on CD45RA expression on Tcyt and Th. Memory cells population was presented as CD3+CD4+CD45RA– for Th, CD3+CD8+CD45RA– for Tcyt. To study memory cell compartments and differentiation stages of Th and Tcyt, we analyzed co-expression of CD45RA and CD62L at their surface. Th and Tcyt cells were separated to ‘naïve’ (CD45RA+CD62L+), central memory (CM, CD45RA–CD62L+), effector memory (EM, CD45RA–CD62L−), and terminally differentiated CD45RA-positive effector memory (TEMRA, CD45RA+CD62L−) cells (Figure 2 and Figure 3).

AS+CD and AS groups demonstrated lower frequency of Th memory cells compared to CD cohort patients (44.06% (39.22; 53.23) and 42.09% (37.81; 58.76) vs. 57.44% (48.34; 66.29) with *p* = 0.008 and *p* = 0.028, respectively). Absolute Th memory cell values differed only between AS and CD cohorts (154 cells/1 μL (102; 255) vs. 315 cells/1 μL (227; 516) with *p* = 0.02). Most prominent differences were observed within the EM compartment. The frequency of EM Th was significantly lower in the AS+CD group compared to the CD-only group (28.13% (15.47; 36.52) vs. 29.82% (24.99; 37.02), *p* = 0.022). In contrast, the absolute count of EM T-helper cells was lower in the AS group than in the CD group (64 cells/μL (36; 118) vs. 132 cells/μL (103; 216), *p* = 0.023) (Figure 2).

Tcyt memory cells frequency in AS+CD was lower compared to CD (12.34% (9.57; 23.58) vs. 25.10% (19.58; 34.15) with *p* = 0.025). Absolute value of Tcyt memory cells in AS+CD cohort and AS was lower, than in the CD group (23 cells/1 μL (14; 71) and 44 cells/1 μL (24; 77) vs. 93 cells/1 μL (59; 134) with *p* = 0.015 and *p* = 0.022, respectively). The most notable differences were also observed within the EM compartment. Both the relative percentages and absolute counts of EM Tcyt were elevated in the AS+CD and AS groups relative to the CD group. For relative values, the AS+CD and AS groups showed 9.98% (6.73; 16.47) and 12.68% (6.82; 16.85), respectively, versus 18.41% (15.63; 26.46) in the CD group (*p* = 0.02 and *p* = 0.037). For absolute counts, the corresponding values were 16 cells/μL (8; 53) and 31 cells/μL (14; 54) compared to 68 cells/μL (52; 110) in the CD group (*p* = 0.028 and *p* = 0.01). Furthermore, the absolute numbers of CM Tcyt and TEMRA Tcyt were significantly reduced in the AS+CD group when compared with the CD group. Specifically, CM counts were 6 cells/μL (4; 18) versus 18 cells/μL (10; 29), and TEMRA counts were 52 cells/μL (33; 136) versus 118 cells/μL (68; 188), with *p*-values of 0.045 and 0.04, respectively (Figure 3).

**Figure 4:**
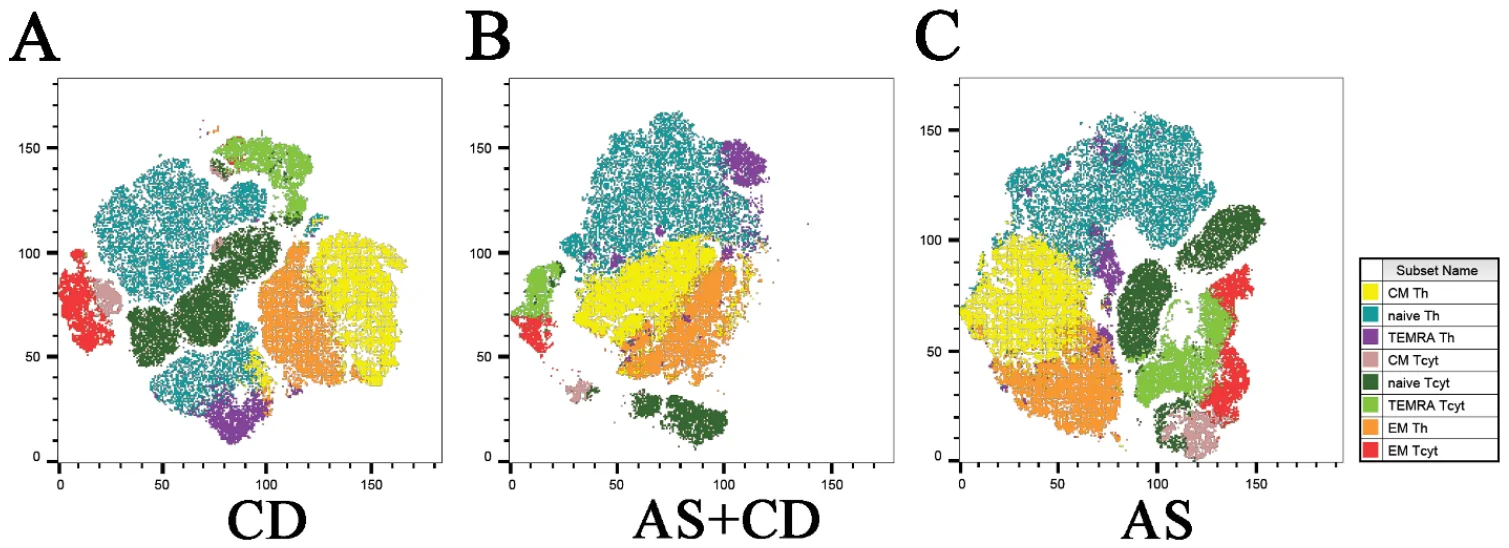
tSNE map of Th and Tcyt maturation subsets in patients with CD, AS+CD, and AS. Panel (**A**) shows tSNE of concatenated data from 16 patients with Crohn’s disease (CD); panel (**B**) shows tSNE of concatenated data from 13 patients with ankylosing spondylitis associated with CD (AS+CD); panel (**C**) shows tSNE of concatenated data from 13 patients with ankylosing spondylitis alone (AS). Co-expression of CD45RA and CD62L was analyzed on Th and Tcyt cells, allowing the identification of four maturation subsets: ‘naïve’ cells (CD45RA+CD62L+), central memory cells (CM, CD45RA–CD62L+), effector memory cells (EM, CD45RA–CD62L–), and TEMRA cells (CD45RA+CD62L–). Surface marker distribution is depicted on the tSNE maps, with relative antigen expression visualized by color intensity (as shown in the legend in the upper right corner of Figure 4).

To identify hidden phenotypic relationships between cell populations, we applied t-Distributed Stochastic Neighbor Embedding (tSNE). This dimensionality reduction method was chosen over others because it prioritizes the preservation of local structure, facilitates the detection of minor cell subsets, and carries a low risk of pseudoclustering. In the resulting plots, EM Tcyt and TEMRA Tcyt cells clustered together in the AS+CD and AS groups, whereas in the CD group, these two populations occupied distinct clusters (Figure 4).

### 3.3. Imbalance of ‘Polarized’ Subsets Within Memory Compartment of Th Cells in Patients with AS+CD

To assess distribution of polarized Th subsets, we used multicolor staining for the chemokine receptors CXCR5, CXCR3, CCR4, and CCR6, following a previously described approach [18]. This strategy allowed us to identify the following cell populations: Th1 (CXCR5–CCR6–CXCR3+CCR4–), Th2 (CXCR5–CCR6–CXCR3–CCR4+), total Th17 (CXCR5–CCR6+), and Tfh (CXCR5+). Sequential gating further enabled the identification of Th17 and Tfh subpopulations, including ‘classical’ CCR6+CCR4+ Th17 cells, ‘non-classical’ CCR6+CXCR3+ Th17.1 cells, double-negative (DN) Th17 cells lacking both CXCR3 and CCR4, double-positive (DP) Th17 cells co-expressing CXCR3 and CCR4, as well as Tfh1 (CXCR3+CCR6–), Tfh2 (CXCR3–CCR6–), Tfh17 (CXCR3–CCR6+), and double-positive DP Tfh (CXCR3+CCR6+). 

**Figure 5:**
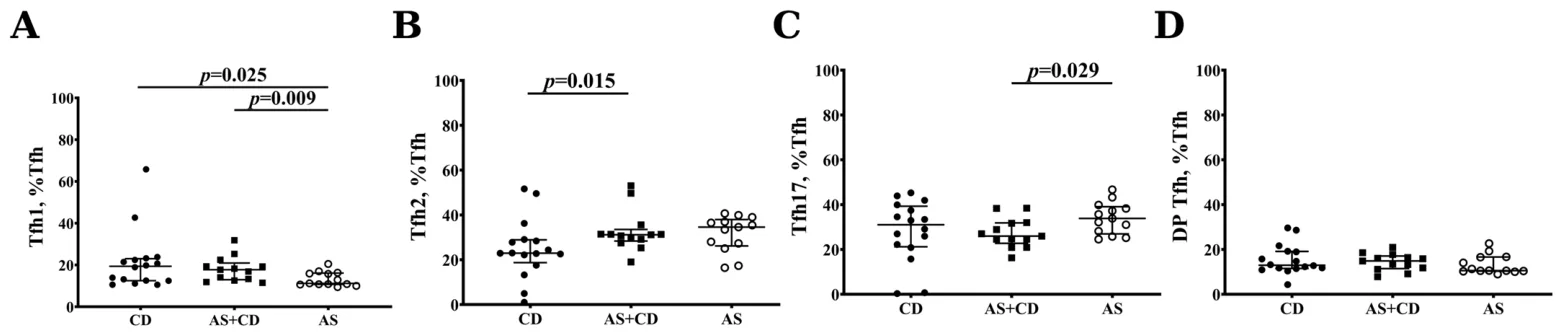
Differences in Tfh subpopulations among the three groups. Scatter plots (**A**–**D**) show the percentages of Tfh1, Tfh2, Tfh17, and DP Tfh cells. Black circles represent patients with Crohn’s disease (CD, n = 16); black squares represent patients with AS associated with CD (AS+CD, n = 13); open circles represent patients with ankylosing spondylitis alone (AS, n = 13). Each symbol corresponds to an individual subject. Horizontal lines indicate the median and interquartile range (Med (Q25; Q75)). Statistical comparisons were performed using the Mann–Whitney U test.

The overall frequencies of Th1, Th2, total Th17, classical Th17, Th17.1, DN Th17, DP Th17, and Tfh did not differ significantly among the three groups (Supplementary Table S4). However, the Tfh1 subpopulation was significantly lower in the AS group compared with both the CD group (11.19% (10.69; 16.09) vs. 19.35% (12.45; 22.99), *p* = 0.025) and the AS+CD group (11.19% (10.69; 16.09) vs. 17.73% (12.95; 20.92), *p* = 0.009). The frequency of Tfh2 was higher in the AS+CD group than in the CD group (31.14% (28.32; 33.50) vs. 22.95% (18.69; 28.86), *p* = 0.015). In turn, the Tfh17 frequency in AS+CD patients was lower than in AS patients (25.95% (22.72; 31.84) vs. 33.81% (26.94; 39.05), *p* = 0.029). The DP Tfh subpopulation showed no significant differences between the groups (Figure 5).

To characterize T-cell differentiation and development as a response to autoantigen and cytokine environment, we measured Th ‘polarization’ within CM and EM compartments. The CM compartment demonstrated a significant increase in Tfh1 concentration in AS+CD in comparison to the AS group. The AS+CD patient cohort demonstrated a decrease in classical Th17 and an increase in DP Tfh within the EM Th compartment when compared to AS. The CD group, however, demonstrated a decreased concentration of EM classical Th17 and EM Tfh17 versus the AS group (Table 2).

### 3.4. Treg CD39 and CD73 Expression Shift in AS+CD Patients

To assess Treg differentiation, we analyzed the surface expression of CD62L and CD45RA, which allowed the identification of four Treg populations: ‘naïve’ Treg (CD45RA+CD62L+), CM Treg (CD45RA–CD62L+), EM Treg (CD45RA–CD62L–), and TEMRA Treg (CD45RA+CD62L–). Comparisons of these populations across the patient groups revealed no statistically significant differences (Supplementary Table S5).

**Figure 6:**
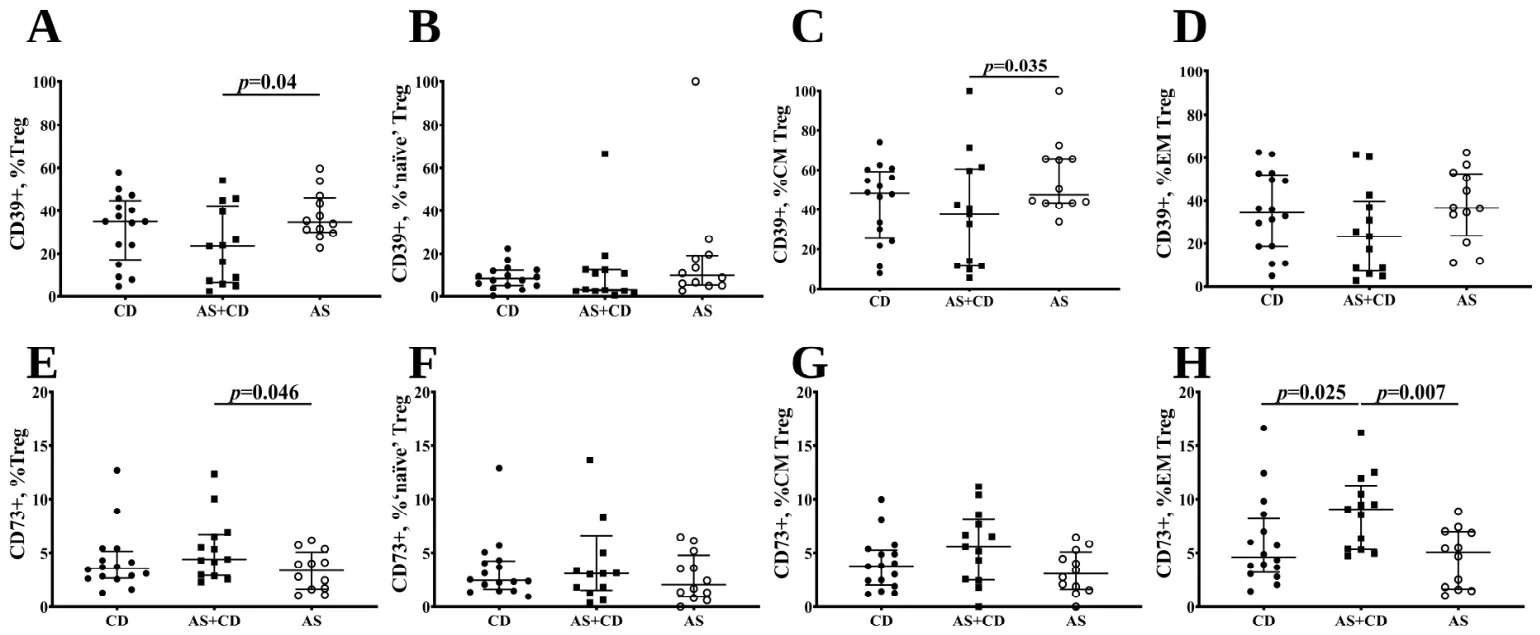
Expression of CD39 and CD73 on different Treg populations in patients with AS+CD. Scatter plots (**A**–**D**) show the percentages of CD39-expressing cells within total Treg, ‘naïve’ Treg, CM Treg, and EM Treg populations. Scatter plots (**E**–**H**) show the percentages of CD73-expressing cells within total Treg, ‘naïve’ Treg, CM Treg, and EM Treg populations. Black circles represent patients with Crohn’s disease (CD, n = 16); black squares represent patients with AS associated with CD (AS+CD, n = 13); open circles represent patients with ankylosing spondylitis alone (AS, n = 13). Each symbol corresponds to an individual subject. Horizontal lines indicate the median and interquartile range (Med (Q25; Q75)). Statistical comparisons were performed using the Mann–Whitney U test.

We next characterized the adenosine-mediated immunosuppressive mechanism in AS+CD patients by analyzing the expression of the ectonucleotidases CD39 and CD73 on the surface of various Treg subpopulations. The frequency of total CD39+ Treg was lower in the AS+CD group compared with the AS group (23.56% (6.66; 42.29) vs. 34.71% (30.15; 46.01), *p* = 0.04) (Figure 6A). In contrast, the concentration of total CD73+ Treg was higher in AS+CD patients than in AS patients (4.41% (2.89; 6.77) vs. 3.35% (1.65; 5.06), *p* = 0.046) (Figure 6E). Furthermore, analysis of CD39 and CD73 on Tregs at different differentiation stages revealed a reduced relative frequency of total CD39+ CM Treg in the AS+CD group compared with the AS group (37.65% (11.41; 60.51) vs. 47.43% (43.38; 65.56), *p* = 0.035) (Figure 6C). Interestingly, the total CD73+ EM Treg population was elevated in AS+CD patients relative to both CD and AS patients (9.09% (5.35; 11.21) vs. 4.61% (3.18; 8.22) and 5.06% (1.65; 7.03), with *p* = 0.025 and *p* = 0.007, respectively) (Figure 6H).

**Figure 7:**
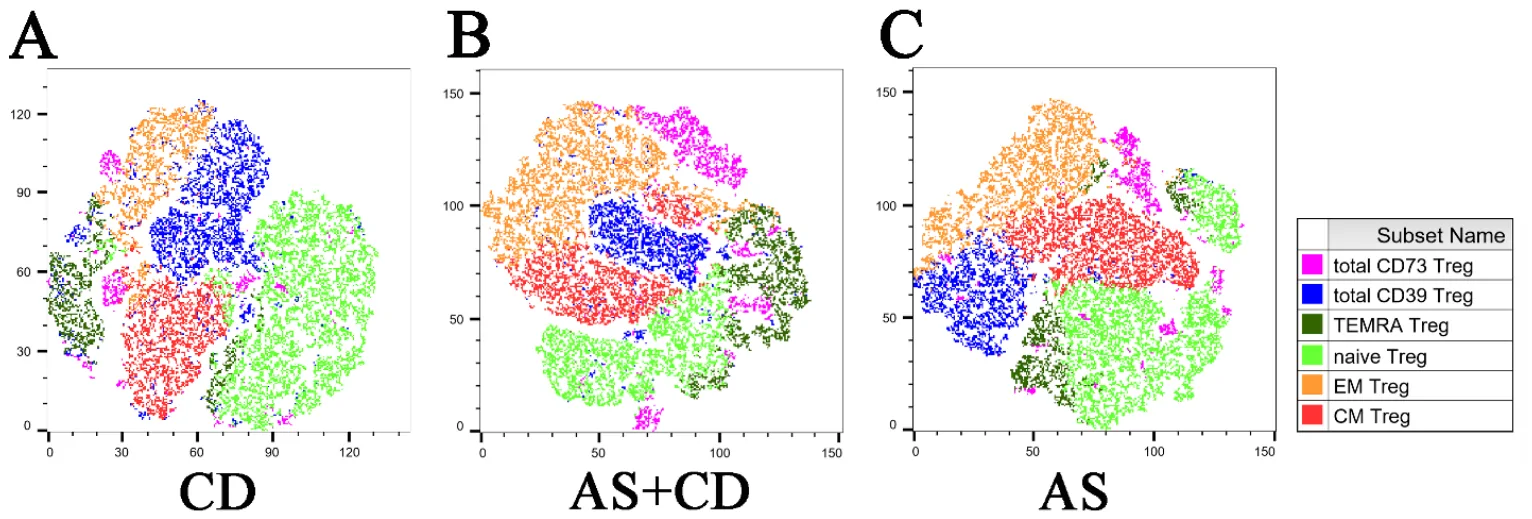
tSNE map of CD39 and CD73 expression on Treg maturation subsets in patients with CD, AS+CD, and AS. Panel (**A**) shows tSNE of concatenated data from 16 patients with Crohn’s disease (CD); panel (**B**) shows tSNE of concatenated data from 13 patients with ankylosing spondylitis associated with CD (AS+CD); panel (**C**) shows tSNE of concatenated data from 13 patients with ankylosing spondylitis alone (AS). Co-expression of CD45RA and CD62L was analyzed on Treg, allowing the identification of four maturation subsets: ‘naïve’ cells (CD45RA+CD62L+), central memory cells (CM, CD45RA–CD62L+), effector memory cells (EM, CD45RA–CD62L–), and TEMRA cells (CD45RA+CD62L–). Surface marker distribution is depicted on the tSNE maps, with relative antigen expression visualized by color intensity (as shown in the legend in the upper right corner of Figure 7).

The tSNE maps constructed for the three groups revealed topographic differences in the density distribution of CD39 and CD73 across the various Treg populations (Figure 7).

### 3.5. Impaired Humoral Compartment of Adaptive Immunity in Patients with AS+CD

Several phenotyping approaches were used to analyze peripheral blood B cells. The first approach, based on IgD and CD38 expression, allowed the identification of ‘naïve’ IgD+CD38– Bm1 cells, ‘activated naïve’ Bm2 cells (IgD+CD38+), pre-germinal-center Bm2′ cells (IgD+CD38++), a common subset comprising centroblasts and centrocytes (designated as Bm3+Bm4 cells, IgD–CD38++), as well as early memory and resting memory cells (eBm5 and Bm5 cells with the phenotypes IgD–CD38+ and IgD–CD38–, respectively). The second approach, based on CD27 and CD38 expression, identified transitional B cells (CD27–CD38++), mature ‘naïve’ B cells (CD27–CD38+), mature activated B cells (CD27+CD38+), plasmablasts (CD27++CD38++), resting memory B cells (CD27+CD38–), and double-negative B cells (CD27–CD38–). The third approach, based on IgD and CD27 expression, identified ‘naïve’ B cells (IgD+CD27–), ‘unswitched’ memory B cells (IgD+CD27+), ‘switched’ memory B cells (IgD–CD27+), double-negative B cells (IgD–CD27–), and plasmablasts (IgD−CD27++). The gating strategy was described by us earlier [18]. Among all populations examined, AS+CD patients showed reduced concentrations of Bm5 and ‘switched’ memory B cells compared with CD patients (Table 3).

**Figure 8:**
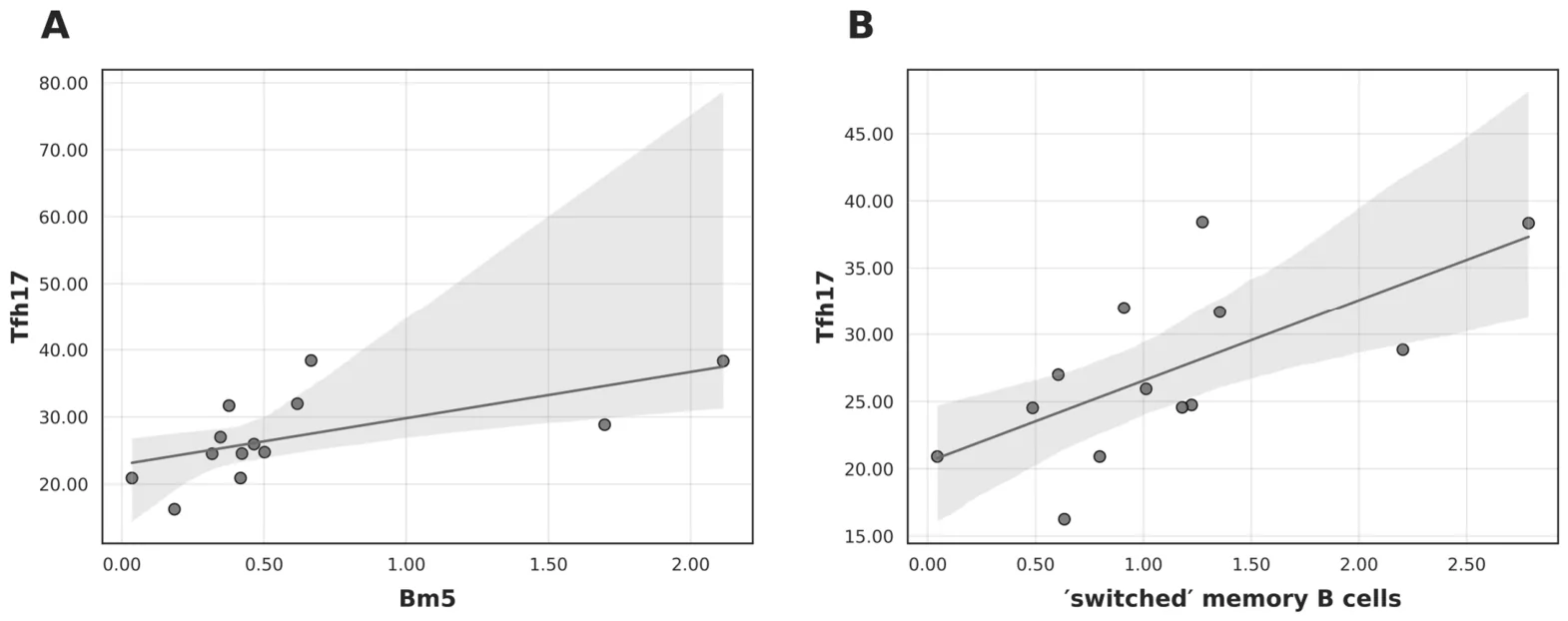
Correlations between Tfh17 and B-cell populations. Panel (**A**) shows the Spearman correlation coefficient between Tfh17 concentration and Bm5 cells; panel (**B**) shows the Spearman correlation coefficient between Tfh17 concentration and ‘switched’ memory B cells.

An interesting finding was that both of these populations had a strong positive correlation with the Tfh17 subpopulation (r = 0.775, *p* = 0.002 for Bm5; r = 0.709, *p* = 0.007 for ‘switched’ memory B cells) (Figure 8A,B).

### 3.6. Associations Between Phenotypic Findings and Clinical-Laboratory Disease Parameters

**Figure 9:**
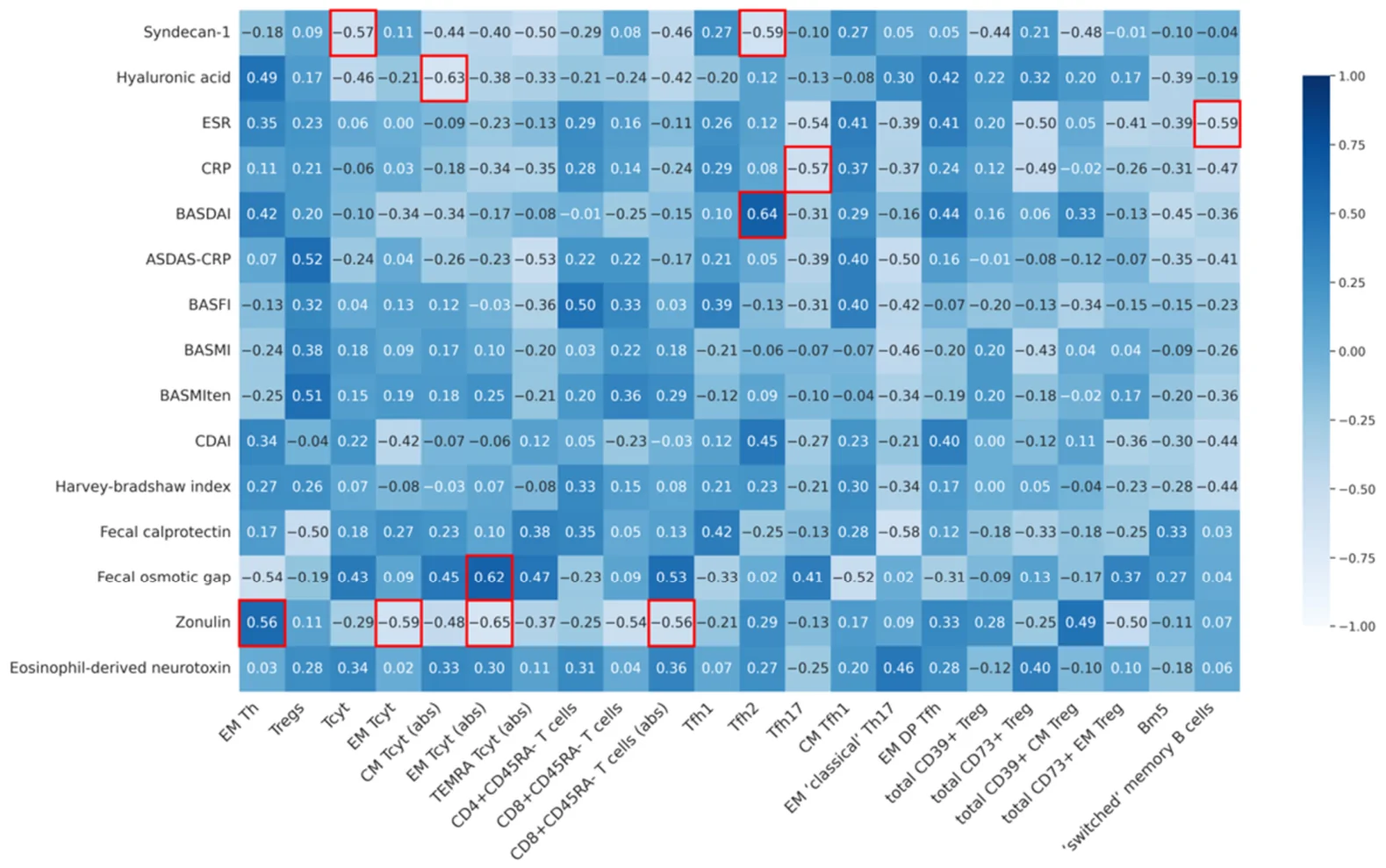
Correlation matrix illustrating the relationships between clinical-laboratory parameters of AS+CD activity and lymphocyte populations. Statistical analysis was performed using Spearman’s rank correlation test. Correlation results are presented as a color gradient, where light blue squares indicate negative correlations and dark blue squares indicate positive correlations. Exact Spearman correlation coefficients are shown as numerical values within each square. Statistically significant correlations (*p* < 0.05) are outlined in red.

To determine the pathogenetic significance of lymphocyte subpopulations that showed the most pronounced intergroup differences in AS+CD patients, we performed correlation analyses with disease activity indices, functional scores, markers of intestinal permeability, and systemic inflammation. Among all activity indices, only BASDAI showed a statistically significant positive correlation with the Tfh2 population. When assessing intestinal markers in the AS+CD group, we observed positive correlations between fecal osmotic gap and the absolute count of EM Tcyt, as well as negative correlations between Syndecan-1 and Tcyt and Tfh2 populations; between Hyaluronic acid and the absolute count of CM Tcyt; and between Zonulin and the EM Tcyt population (both relative and absolute counts) and the absolute count of CD8+CD45RA– T cells. Analysis of correlations between lymphocyte subpopulations and acute-phase markers (C-reactive protein (CRP) and erythrocyte sedimentation rate (ESR)) revealed negative correlations between CRP and Tfh17, and between ESR and ‘switched’ memory B cells (Figure 9).

### 3.7. Comparison of Identified Lymphocyte Populations Across the Three Patient Groups and Healthy Controls

**Figure 10:**
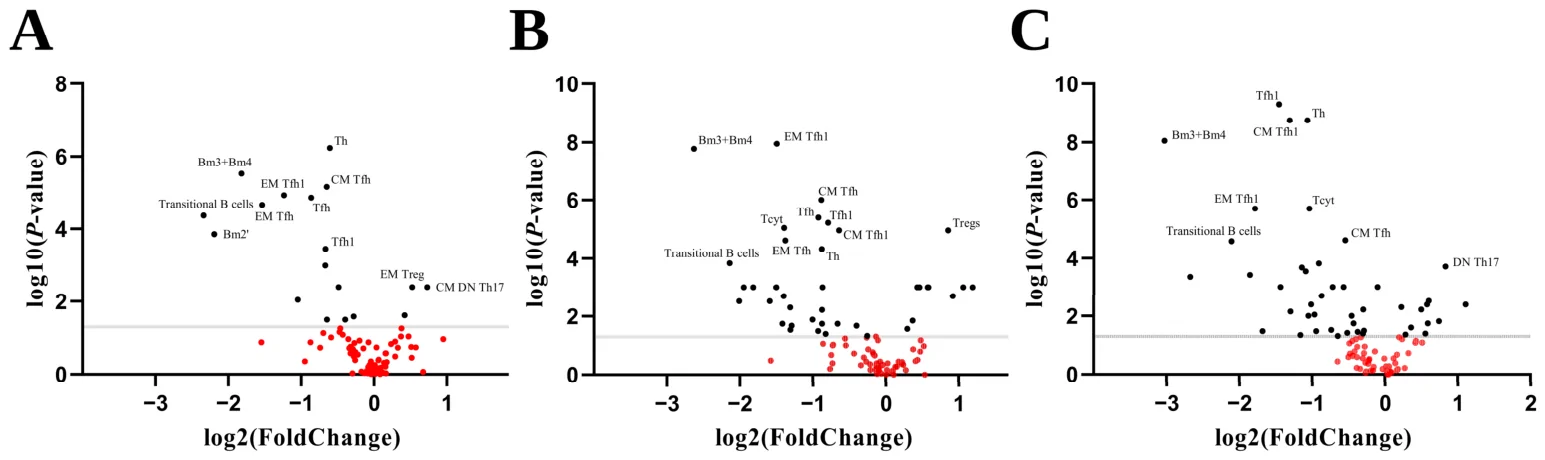
Volcano-plots illustrating the 94 ranked T- and B-cell subsets count changes between patients with CD, AS+CD, AS, and HC. Volcano-plot (**A**) illustrating the 94 ranked T- and B-cell subset count changes between patient with Crohn’s disease (n = 16) and healthy controls (n = 26); Volcano-plot (**B**) illustrating the 94 ranked T- and B-cell subsets count changes between patient with ankylosing spondylitis associated with Crohn’s disease (n = 13) and healthy controls (n = 26); Volcano-plot (**C**) illustrating the 94 ranked T- and B-cell subset count changes between patient with ankylosing spondylitis (n = 13) and healthy controls (n = 26). The −log10 corrected *p*-value is plotted against the log2 fold change. Red coloring points below the line denote *p* > 0.05, which is our significance threshold (prior to logarithmic transformation). Statistical significance was assessed using Mann–Whitney U test.

To assess the degree of immune dysregulation in patients with CD, AS+CD, and AS, we compared all three groups with healthy controls. The most pronounced differences between the patient groups and healthy controls are presented in Figure 10. In CD patients, compared with healthy donors, we observed reduced concentrations of Th cells, Bm3+Bm4 cells, CM Tfh, EM Tfh1, Tfh, EM Tfh, transitional B cells, Bm2′ cells, and Tfh1, whereas EM Treg and CM DN Th17 levels were increased (Figure 10A). In the AS+CD group, circulating levels of EM Tfh1, Bm3+Bm4, CM Tfh, Tfh, Tfh1, Tcyt, CM Tfh1, EM Tfh, Th cells, and transitional B cells were decreased, while Treg concentrations were elevated (Figure 10B). Comparison of lymphocyte populations between AS patients and healthy controls revealed reduced frequencies of Tfh1, Th cells, CM Tfh1, Bm3+Bm4, EM Tfh1, Tcyt, transitional B cells, and CM Tfh, whereas DN Th17 levels were increased (Figure 10C).

## 4. Discussion

### 4.1. Key Observations

This study is the first systematic evaluation of peripheral blood T- and B-cell subset composition in patients with AS associated with CD compared with patients with AS alone and patients with CD alone. A distinctive feature of this study is the patient cohort: all groups included individuals with criterion-verified diagnoses and active AS and CD.

The elevated peripheral blood Treg frequency observed in patients with ankylosing spondyloarthritis associated with Crohn’s disease probably mirrors the magnitude of inflammatory activity across multiple affected sites, including the axial skeleton, peripheral joints, and the intestinal tract.

Tregs can be induced in peripheral lymphoid organs in response to various antigens (autoantigens in the case of autoimmune diseases) and the local microenvironment (predominantly IL-2 and TGF-β) [19,20]. In AS associated with CD, potential autoantigens include immunomodulatory molecules such as CD74 [21], proteins regulating bone metabolism (sirtuin-1, chondromodulin 1, osteoglycin, osteonectin, sclerostin, noggin) [22,23,24], and proteins sharing common epitopes with bacterial peptides (PRPF3, GPER1, and RNASEH2B) [25]. Treg induction may also occur as a result of T-cell receptor (TCR) recognition of commensal microbiota antigens and dietary antigens, given that intestinal barrier permeability is impaired in AS associated with CD [26]. The cytokines required for differentiation may be produced by activated T cells (IL-2), tolerogenic dendritic cells (TGF-beta), or released from damaged cells as a consequence of tissue inflammation [27]. Furthermore, Tregs may potentially be activated in a TCR-independent manner via cytokine signaling and Toll-like receptor stimulation (so-called «bystander» T-cell activation) [28]. Given the presence of inflammation localized in multiple organ systems in AS associated with CD, Treg induction occurs to a greater extent than in AS and CD alone.

An imbalance of Tregs expressing the ectonucleotidases CD39 and CD73 on their surface was observed predominantly in the memory compartments (effector and central memory) in patients from the AS+CD group. These molecules on the Treg surface are required for the breakdown of pro-inflammatory extracellular adenosine triphosphate (ATP) into anti-inflammatory adenosine. CD39 hydrolyzes ATP to adenosine monophosphate (AMP), and CD73 then cleaves AMP to adenosine. Adenosine interacts with four G-protein-coupled receptors (A1, A2A, A2B, and A3), each of which has distinct affinity and cell-specific effects [29]. The A2A receptor is expressed on the surface of Th17 cells, macrophages, and dendritic cells. Upon binding with adenosine, this receptor reduces Th17 differentiation and IL-17 synthesis, while promoting macrophage polarization toward the M2 phenotype and increasing production of the anti-inflammatory cytokine IL-10 [30]. In AS associated with CD, purinergic homeostasis may be disrupted, and CD39 and CD73 levels on Treg surfaces may change in opposite directions as a result of active inflammation and metabolic adaptation. It is plausible that, similar to what is observed in CD alone, the levels of CD39-specific antisense RNA are increased in Tregs from patients with AS+CD, which suppresses CD39 expression [31,32]. In AS, CD39 expression is also reduced due to chronic exposure to IL-6, IL-17, and TNF-alpha [30]. Collectively, these data may explain the significant reduction in the number of CD39+ Tregs in patients with AS associated with CD. Furthermore, chronic inflammation induces hypoxia, promoting Tregs to express hypoxia-inducible factor 1-alpha (HIF1A) and shift their metabolism toward aerobic glycolysis, which is accompanied by increased CD73 expression [33]. Therefore, in AS associated with CD, the downregulation of CD39 on Tregs may be related to the presence of antisense RNA and chronic production of pro-inflammatory cytokines. In turn, the increased expression of CD73 is likely associated with pronounced metabolic reprogramming induced by hypoxia.

Additionally, AS associated with CD patients exhibited reduced Tcyt counts compared with both CD patients and healthy controls. Moreover, this population showed a moderate negative correlation with syndecan-1. Syndecan-1 is involved in maintaining intestinal barrier integrity. In IBD, elevated syndecan-1 levels in peripheral blood reflect intestinal barrier disruption resulting from autoimmune inflammation [34]. It is possible that the reduction of this population in the AS+CD group is related to their migration into inflammatory foci located predominantly in the joints and entheses, rather than into the intestinal mucosal lining. Further analysis of Tcyt subpopulations revealed a decrease in EM Tcyt in the AS+CD and AS groups, and this cell population clustered together with TEMRA Tcyt on the tSNE map (Figure 3 and Figure 4). No such changes were observed in the CD group. These findings may suggest an arthritogenic role of effector Tcyt populations, particularly EM Tcyt, in patients with AS associated with Crohn’s disease. Our hypothesis is consistent with recent literature providing evidence for the central role of Tcyt cells in recognizing arthritogenic peptides presented on HLA-B27 major histocompatibility complex class I molecules in patients with SpA [35,36].

We also identified an imbalance in memory Th cells in the AS+CD and AS groups, driven mainly by a decrease in EM Th. Furthermore, in the AS+CD group, the EM Th population showed a positive correlation with zonulin. Zonulin functions as a regulator of intestinal barrier permeability. This protein activates PAR-2 and EGFR receptors, leading to rearrangement of the actin cytoskeleton and displacement of tight junction proteins such as zonula occludens-1 (ZO-1), thereby increasing intestinal wall permeability [37,38]. The above-mentioned mechanism is required to allow passage of a limited number of antigens in order to maintain the balance between local immune response and the development of tolerance to commensal microbiota [37,39]. In IBD, zonulin serves as a marker of intestinal permeability and reflects the severity of intestinal wall inflammation [40]. Therefore, it is plausible that EM Th populations migrate to the inflammatory foci in the gut, where they contribute to the development of autoimmune inflammation. Of note is the reduced number of ‘classical’ Th17 cells in the effector memory compartment. According to the current literature, under the influence of pro-inflammatory cytokines such as IL-23, IL-12, IL-1β, TNF-α, and IFN-γ, these cells may shift their phenotype toward pathogenic Th17.1 cells, which are involved in the development and maintenance of intestinal wall inflammation [14,41,42]. In AS associated with CD, an increase in the Tfh2 population was observed compared with CD. Furthermore, in patients with AS and AS+CD, but not in those with CD, this population was elevated in peripheral blood relative to healthy controls. Additionally, in the AS+CD group, Tfh2 showed a positive correlation with the BASDAI activity index. Tfh2 cells are known to promote class switching of antibody production predominantly toward IgE and IgG4 [43]. IgE is primarily involved in allergic reactions, whereas IgG4 is associated with fibrosis development [44,45,46]. Recent studies have demonstrated the presence of IgG4+ B cells at sites of inflammation in AS patients [47]. Thus, in AS associated with CD patients, the increase in circulating Tfh2 cells may be driven by chronic inflammation and may contribute to fibrotic tissue development and the formation of ankyloses.

Comparative analysis across groups revealed opposing directional changes in Tfh1 cell frequencies among patients with AS associated with CD. According to the literature, Tfh1 cells have a lower capacity to stimulate B cells compared to Tfh2 and Tfh17 cells [48]. Moreover, this population predominantly performs regulatory functions, including suppression of spontaneous and excessive B-cell proliferation [49]. An imbalance between Tfh1 cells on the one hand and Tfh2 and Tfh17 cells on the other has been described in various autoimmune diseases, including systemic lupus erythematosus [50], primary Sjögren’s syndrome (pSS) [51], IgG4-Related Disease (IgG4-RD) [52], psoriasis vulgaris [53], and lung sarcoidosis [18]. Their function is impaired in autoimmune diseases, which may explain their reduced numbers in all three patient groups compared to the control group. The milder reduction seen in the AS+CD and CD groups could indicate a requirement for a selective B-cell response directed against bacterial antigens at the intestinal mucosal surface. This hypothesis is supported by the changes we observed in Tfh17 and ‘switched’ memory B-cell populations in AS+CD patients. In AS associated with CD, the Tfh17 population was more markedly reduced compared with AS. Similarly, ‘switched’ memory B cells in AS+CD patients were decreased relative to both CD patients and healthy donors. We found strong positive correlations between Tfh17 and ‘switched’ memory B cells; moreover, these populations correlated negatively with the acute-phase markers CRP and ESR, respectively (Figure 7B and Figure 8). Therefore, it is plausible that Tfh17 and ‘switched’ memory B cells migrate to inflammatory foci in the intestinal mucosa, where they contribute to the production of secretory IgA for opsonization and reduction of opportunistic microorganism translocation (so-called «immune exclusion») [54]. We therefore hypothesize that, in AS+CD, Tfh17 and ‘switched’ memory B cells play an anti-inflammatory role at the level of the intestinal wall.

**Figure 11:**
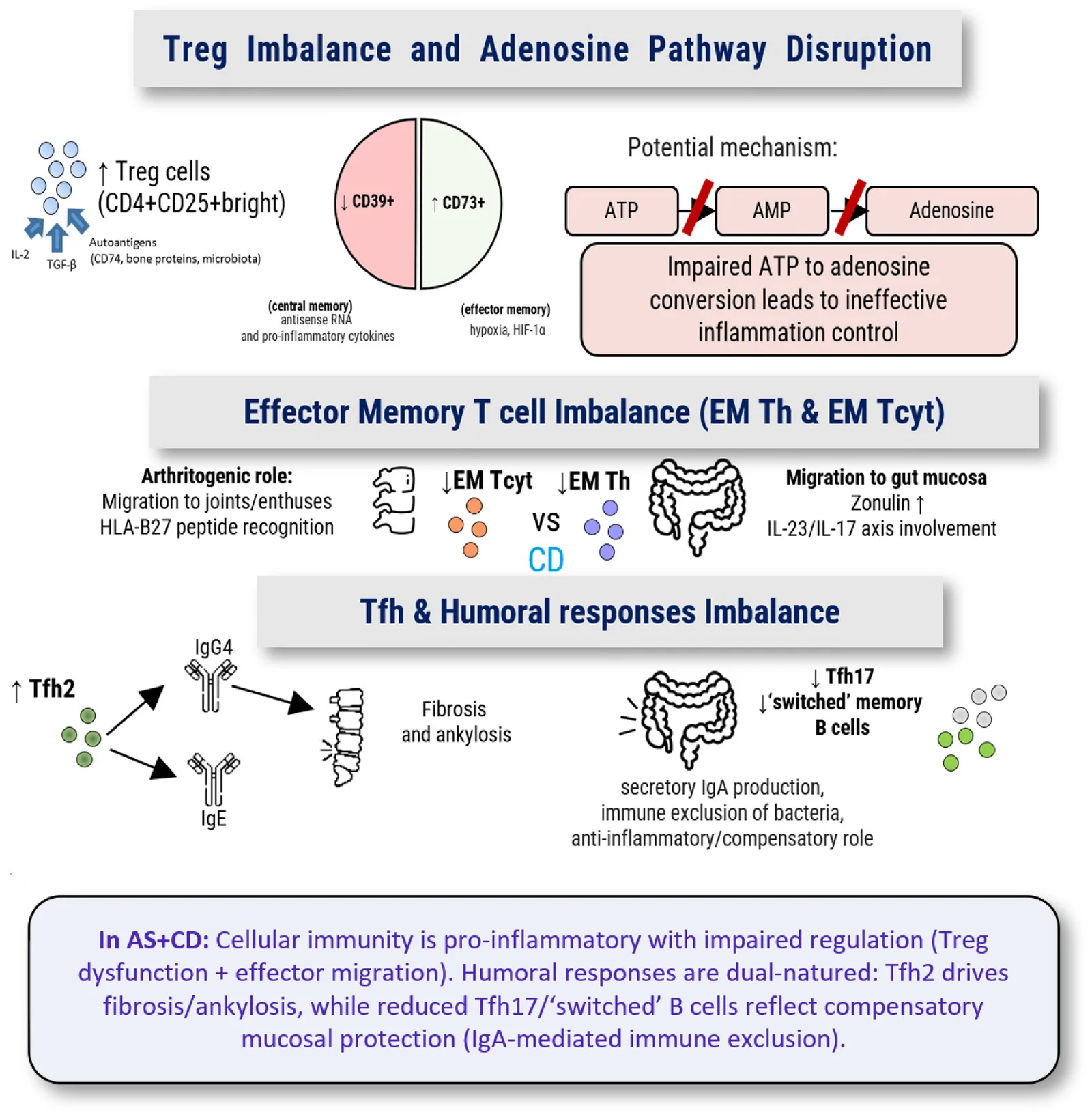
Schematic illustration of T and B cell subset changes in ankylosing spondylitis associated with Crohn’s disease. Abbreviations for Figure 11: Treg—regulatory T cells; EM Th—effector memory T-helper cells; EM Tcyt—effector memory cytotoxic T cells; Tfh—T-follicular helper cells; AS+CD—ankylosing spondylitis associated with Crohn’s disease.

The key findings are summarized in Figure 11.

### 4.2. Comparison with Existing Literature Data

Over the last five years, only a few studies have investigated peripheral blood lymphocyte populations in patients with SpA associated with IBD, and even fewer have focused specifically on AS associated with CD. A recently published study by Luo et al. examined peripheral blood T- and B-cell phenotypes in AS patients with gut inflammation [55]. That study included a total of 50 AS patients, comprising individuals with AS without intestinal involvement, AS with colorectal inflammation, and AS with terminal ileitis. The authors found that in patients with intestinal inflammation, the total T cells and CD4+ T-cell pools were increased, whereas Th2 and Tcyt cells were significantly reduced. In AS patients with terminal ileitis, an increase in the total B-cell pool and ‘switched’ memory B cells was observed. In contrast, the Tfh1, Tfh2, and Tfh17 populations did not differ significantly between AS patients with and without gut inflammation [55].

Our findings do not align with these data. In our AS associated with CD patients, we observed no differences in the levels of total T cells, Th cells, Th2 cells, Tcyt cells, or the total B-cell pool compared with AS patients. Furthermore, we found increased Tfh1 concentrations and decreased Tfh17 levels in the AS+CD group relative to AS. In turn, ‘switched’ memory B cells differed significantly only between the AS+CD and CD groups in our cohorts. These discrepancies may be primarily attributable to differences in the clinical characteristics of the patient populations studied. In our investigation, both diseases in the AS+CD cohort were criterion-verified and exhibited clinical and laboratory activity. Additionally, the observed divergences may relate to differences in therapeutic approaches applied to the study groups, as the Luo et al. study reported no correlations between cell populations and disease activity [55]. It should be noted that this study also did not include a comparison between the group of interest and a group with gut inflammation without AS.

Another research group conducted a detailed analysis of T cells in patients with Crohn’s disease-associated axial spondyloarthritis (CD-axSpA) [56]. Lefferts et al. found increased numbers of Th and Tcyt cells expressing granzyme B in CD-axSpA patients. In addition, this group showed reduced ‘naïve’ Th and ‘naïve’ Tcyt cells, accompanied by an increase in EM Th cells. The authors also noted elevated plasma IFN-gamma levels in a subset of CD-axSpA patients [56]. These findings are not consistent with our data. We observed no significant differences in ‘naïve’ Th or ‘naïve’ Tcyt levels among the three groups, whereas EM Th levels in the AS+CD group were significantly decreased compared with CD patients. This discrepancy may be explained by the absence of active CD in the patient cohort studied by those authors (all study groups had an SCCAI activity index below 4.5 points), whereas our CD and AS+CD groups had active CD.

Earlier studies of peripheral blood in AS patients with gut involvement demonstrated an expansion of Tregs compared with healthy controls, which is consistent with our findings, as AS associated with CD patients had increased Treg counts relative to healthy donors [57].

As noted above, few investigators have studied peripheral blood T- and B-cell populations in patients with AS associated with CD. This is likely due primarily to challenges in patient recruitment (the small number of such patients and difficulties in assembling a clinically homogeneous cohort) and in selecting the most appropriate study design. Nevertheless, investigation of this spondyloarthritis phenotype is important for elucidating the key pathogenic mechanisms of the disease and for developing optimal patient management strategies.

### 4.3. Study Limitations

This study had several limitations. The main limitations of our study are the small sample size, the heterogeneity of the groups, and the lack of stringent statistical corrections. For between-group comparisons, we did not apply multiple testing corrections due to the small sample sizes and the presumably small effect sizes. Therefore, the borderline *p*-values obtained should be interpreted with caution and require further validation in independent cohorts. Moreover, multivariable analysis adjusting for age, sex, disease duration, disease activity, biological treatment, corticosteroid therapy, HLA-B27 status, intestinal disease phenotype, musculoskeletal phenotype, and treatment effects was not performed due to the limited number of patients in the group of interest and the cross-sectional, observational design of the study. Variability in the extent of gastrointestinal involvement in Crohn’s disease, differences in disease activity across all three groups, and the presence of therapy may all potentially contribute to the observed results. The lack of a validation cohort also represents a limitation with respect to the interpretation and extrapolation of our findings.

Furthermore, biological material from pathological sites (intestine, joints, entheses) was not available. Consequently, the immune cell phenotypes observed in peripheral blood may only partially reflect the immunological processes occurring at the sites of disease. In addition, no in vitro functional assays were performed, which limits our conclusions regarding the functional activity of the identified cell populations.

It should be noted that flow cytometric analysis of Th17 and Treg populations was performed with the use of indicative markers. For Th17 identification, CD161 expression was not assessed. Likewise, CD127 expression on CD4^+^ T cells was not evaluated for Treg identification.

### 4.4. Future Perspectives for Clinical Practice

The preliminary data obtained in this pilot study define directions for subsequent analysis of T- and B-cell populations in larger cohorts. In future large-scale validation studies of peripheral blood lymphocyte phenotypes, immunological markers may be identified that could aid in the differential diagnosis of AS associated with CD.

In the early stages of AS, extra-skeletal manifestations often remain undetected, which complicates the selection of the appropriate bDMARD class and delays the initiation of the most effective therapy. A typical example is the difficulty in detecting coexisting IBD: the absence of clinical symptoms, ambiguous endoscopic findings at early stages, and false-positive fecal calprotectin results due to background NSAID therapy all contribute to delayed diagnosis and, consequently, delayed initiation of effective treatment [58]. Equally challenging is the differentiation between inflammatory spinal involvement as an extra-intestinal manifestation of CD and true AS associated with CD [59]. Longitudinal studies aimed at identifying predictors of CD development in axial spondyloarthritis and predictors of axial spondyloarthritis development in CD would help guide the management of these patients.

Furthermore, expansion of the cohort and further studies of patients with AS associated with CD would enable the identification of a specific immune cell phenotype that plays a central role in disease development and progression, which may ultimately serve as a basis for the development of targeted therapy directed against this lymphocyte population. Prospective studies of patients with AS associated with CD would facilitate the identification of immunological predictors of response to bDMARD and tsDMARD therapy, thereby aiding in the development of recommendations for initiating the most effective treatment.

## 5. Conclusions

In this pilot study, we identified phenotypic alterations in T and B cells that appear to be most characteristic of the spondyloarthritis phenotype AS associated with CD. Our findings suggest that the alterations in T cell-mediated adaptive immunity may be of a chronic pro-inflammatory nature, potentially accompanied by some degree of exhaustion of the regulatory component. The changes observed in the humoral component of the adaptive immune response in this condition might be compensatory in nature and could reflect an attempt to resolve inflammation. Further large-scale investigations of the phenotypic characteristics of peripheral blood lymphocytes in patients with AS associated with CD are needed to gain a deeper understanding of the autoimmune reactions that may underlie the pathogenesis of this disease.

## Figures and Tables

**Table 1:** The characteristics of patients with CD, AS associated with CD, and AS.

Parameter	CD	AS+CD	AS
Disease Duration, years	8.5 (3.0; 13.5)	9 (4; 18)	9 (4; 13)
Sex	M 43.8% (7/16), F 56.3% (9/16)	M 53.8% (7/13), F 46.2% (6/13)	M 76.9% (10/13), F 23.1% (3/13)
csDMARDs	37.5% (6/16)	38.5% (5/13)	0% (0/13)
bDMARDs	62.5% (10/16)	38.5% (5/13)	7.7% (1/13)
NSAIDs	0% (0/16)	15.4% (2/13)	84.6% (11/13)
Corticosteroids	18.8% (3/16)	30.8% (4/13)	7.7% (1/13)
SterRes	6.3% (1/16)	0% (0/13)	0% (0/13)
SterDep	18.8% (3/16)	46.2% (6/13)	0% (0/13)
HLA-B27+	6.3% (1/16)	61.5% (8/13)	92.3% (12/13)
RgSI	–	38.5% (5/13)	61.5% (8/13)
MRSI	–	69.2% (9/13)	69.2% (9/13)
RgSynd	–	23.1% (3/13)	53.8% (7/13)
MRspond	–	23.1% (3/13)	61.5% (8/13)
IBP	–	61.5% (8/13)	61.5% (8/13)
Arthritis	–	38.5% (5/13)	46.2% (6/13)
Enthesitis	–	38.5% (5/13)	30.8% (4/13)
Uveitis	–	15.4% (2/13)	7.7% (1/13)
UlcEros	87.5% (14/16)	76.9% (10/13)	7.7% (1/13)
Inflammatory CD	62.5% (10/16)	84.6% (11/13)	–
Stricturing CD	25.0% (4/16)	15.4% (2/13)	–
Penetrating CD	12.5% (2/16)	0% (0/16)	–
Ileal CD	6.3% (1/16)	30.8% (4/13)	–
Colonic CD	62.5% (10/16)	23.1% (3/13)	–
Ileocolonic CD	25.0% (4/16)	46.2% (6/13)	–
Isolated upper disease CD	6.3% (1/16)	0% (0/13)	–
Perianal involvement	43.8% (7/16)	23.1% (3/13)	–
Surgical treatment	37.5% (6/16)	0% (0/13)	–
CDAI	152.0 (65.3; 225.0)	155.0 (57.0; 228.5)	–
HBI	4.5 (2.0; 9.8)	4.0 (2.0; 9.0)	–
BASDAI	–	4.0 (2.8; 4.4)	3.2 (2.2; 4.8)
ASDAS-CRP	–	2.5 (2.2; 3.2)	3.2 (1.8; 3.8)
BASFI	–	2.6 (0.8; 5.9)	2.1 (0.6; 4.3)
BASMI	–	1 (0; 2)	0 (0; 1)
BASMIten	–	1.8 (1.0; 2.6)	1.6 (1.0; 2.2)
MASES	–	0 (0; 2)	0 (0; 1)

Abbreviation for Table 1: csDMARDs—conventional synthetic disease-modifying antirheumatic drugs; bDMARDs—biological disease-modifying antirheumatic drugs; NSAIDs—non-steroidal anti-inflammatory drugs; SterRes—corticosteroid resistance; SterDep—corticosteroid dependency; RgSI—radiographic sacroiliitis; MRSI—MRI sacroiliitis; RgSynd—radiographic spondylitis; MRspond—MRI spondylitis; IBP— inflammatory back pain; UlcEros—intestinal ulcers and erosions; CDAI—Crohn’s Disease Activity Index; HBI—Harvey–Bradshaw Index; BASDAI—Bath Ankylosing Spondylitis Disease Activity Index; ASDAS-CRP—Ankylosing Spondylitis Disease Activity Score, calculated using C-reactive protein; BASFI—Bath Ankylosing Spondylitis Functional Index; BASMI—Bath Ankylosing Spondylitis Metrology Index; BASMIten—Bath Ankylosing Spondylitis Metrology Index 10-point scale; MASES—Maastricht Ankylosing Spondylitis Enthesis Score.

**Table 2:** Comparison of ‘polarized’ Th populations within the central and effector memory compartments.

	CD (n = 16)	AS+CD (n = 13)	AS (n = 13)	Significant Differences
CM Th1	14.25 (10.4; 17.82)	12.3 (9.86; 20.23)	9.54 (7.72; 15.34)	p_1_ = 0.948 p_2_ = 0.068 p_3_ = 0.05
CM Th2	8.81 (5.45; 16.39)	14.16 (9.39; 15.73)	15.68 (10.48; 18.98)	p_1_ = 0.288 p_2_ = 0.05 p_3_ = 0.223
CM total Th17	36.38 (28.12; 50.17)	42.94 (29.89; 46.31)	44.25 (37.73; 47.44)	p_1_ = 0.914 p_2_ = 0.398 p_3_ = 0.362
CM ‘classical’ Th17	38.37 (22.68; 48.24)	33.93 (29.99; 43.81)	41.73 (35.52; 49.39)	p_1_ = 0.846 p_2_ = 0.215 p_3_ = 0.05
CM Th17.1	21.96 (14.84; 30.18)	26.67 (20.63; 34.92)	21.83 (17.55; 26.32)	p_1_ = 0.329 p_2_ = 0.88 p_3_ = 0.153
CM DN Th17	12.59 (8.62; 16.11)	11.3 (7.62; 14.57)	11.53 (7.6; 14.21)	p_1_ = 0.714 p_2_ = 0.475 p_3_ = 0.88
CM DP Th17	21.09 (18.03; 29.97)	24.74 (21.79; 28.96)	22.76 (18.4; 28.34)	p_1_ = 0.423 p_2_ = 0.914 p_3_ = 0.479
CM Tfh	11.66 (7.09; 14.29)	9.9 (7.41; 11.89)	12.51 (6.78; 14.15)	p_1_ = 0.475 p_2_ = 1 p_3_ = 0.311
CM Tfh1	19.84 (10.88; 24.21)	20.2 (11.98; 24.14)	12.72 (11.14; 17.25)	p_1_ = 0.948 p_2_ = 0.092 p_3_ = 0.044
CM Tfh2	19.21 (14.05; 24.24)	21.5 (17.84; 24.66)	22.72 (14.31; 29.59)	p_1_ = 0.589 p_2_ = 0.65 p_3_ = 0.88
CM Tfh17	30.53 (22.82; 43.06)	29.09 (26.1; 35.63)	35.01 (31.76; 45.75)	p_1_ = 0.914 p_2_ = 0.11 p_3_ = 0.05
CM Tfh DP	14.63 (12.56; 20.32)	19.4 (13.39; 21.69)	16.86 (12.71; 21.19)	p_1_ = 0.374 p_2_ = 0.846 p_3_ = 0.545
EM Th1	17.79 (14.33; 27.79)	14.09 (9.56; 22.44)	16.93 (10.49; 24.65)	p_1_ = 0.17 p_2_ = 0.559 p_3_ = 0.614
EM Th2	1.92 (1.01; 3.05)	1.8 (1.07; 2.73)	2.16 (1.31; 2.81)	p_1_ = 0.983 p_2_ = 0.682 p_3_ = 0.579
EM total Th17	51.74 (47.25; 62.54)	62.16 (44.49; 73.37)	59.19 (54.87; 66.58)	p_1_ = 0.249 p_2_ = 0.101 p_3_ = 0.687
EM ‘classical’ Th17	24.26 (10.27; 32.58)	22.13 (16.65; 30.29)	36.46 (27.39; 41.14)	p_1_ = 0.914 p_2_ = 0.04 p_3_ = 0.007
EM Th17.1	33.4 (26.26; 50.26)	44.87 (27.68; 50.01)	33.61 (32.34; 43.06)	p_1_ = 0.619 p_2_ = 1 p_3_ = 0.362
EM DN Th17	4.96 (3.92; 16.26)	7.96 (5.18; 12.43)	8.24 (5.57; 12.29)	p_1_ = 0.423 p_2_ = 0.503 p_3_ = 1
EM DP Th17	21.32 (17.86; 30.99)	23.17 (18.92; 34.94)	18.55 (16.81; 26.38)	p_1_ = 0.746 p_2_ = 0.184 p_3_ = 0.139
EM Tfh	3.29 (2.34; 5.89)	3.66 (1.59; 5.86)	7.74 (1.99; 8.8)	p_1_ = 0.914 p_2_ = 0.232 p_3_ = 0.204
EM Tfh1	13.01 (8.07; 20.83)	10.85 (5.61; 15.03)	8.88 (2.77; 17.99)	p_1_ = 0.374 p_2_ = 0.288 p_3_ = 0.88
EM Tfh2	17.79 (9.95; 23.59)	20.06 (18.15; 23.86)	20.59 (15.11; 25.36)	p_1_ = 0.17 p_2_ = 0.288 p_3_ = 0.88
EM Tfh17	33.51 (27.96; 39.2)	36.48 (26.14; 41.36)	43.26 (32.84; 50.96)	p_1_ = 0.619 p_2_ = 0.036 p_3_ = 0.153
EM Tfh DP	19.53 (14.36; 26.88)	20.23 (14.02; 26.15)	16.18 (9.38; 19.16)	p_1_ = 0.846 p_2_ = 0.075 p_3_ = 0.026

p_1_—statistical differences between patients with CD and patients with AS associated with CD; p_2_—statistical differences between patients with CD and patients with AS; p_3_—statistical differences between patients with AS associated with CD and patients with AS. The differences between the groups were estimated using Mann–Whitney U-test without correction for multiple testing. The quantitative data are presented as median and quartile ranges (Med (Q25; Q75)).

**Table 3:** Comparison of peripheral blood B-cell subset frequencies among the patient groups.

	CD (n = 16)	AS+CD (n = 13)	AS (n = 13)	Significant Differences
Bm1 (IgD+CD38−)	1.39 (0.87; 2.17)	1.04 (0.41; 1.67)	1.46 (0.81; 3.32)	p_1_ = 0.268 p_2_ = 0.732 p_3_ = 0.168
Bm2 (IgD+CD38+)	7.72 (3.74; 11.36)	7.82 (4.74; 12.15)	6.23 (3.53; 8.74)	p_1_ = 0.88 p_2_ = 0.397 p_3_ = 0.27
Bm2′ (IgD+CD38++)	0.24 (0.1; 0.53)	0.42 (0.05; 0.85)	0.3 (0.17; 0.42)	p_1_ = 0.948 p_2_ = 0.537 p_3_ = 0.936
Bm3+Bm4 (IgD−CD38++)	0.04 (0.01; 0.07)	0.02 (0.01; 0.04)	0.02 (0.01; 0.03)	p_1_ = 0.184 p_2_ = 0.28 p_3_ = 0.769
eBm5 (IgD−CD38+)	0.91 (0.77; 2.14)	0.93 (0.52; 1.09)	0.91 (0.51; 1.14)	p_1_ = 0.249 p_2_ = 0.347 p_3_ = 0.769
Bm5 (IgD−CD38−)	1.02 (0.46; 1.48)	0.42 (0.33; 0.64)	0.62 (0.49; 1.16)	p_1_ = 0.032 p_2_ = 0.28 p_3_ = 0.11
Transitional B cells (CD27−CD38++)	0.22 (0.13; 0.56)	0.26 (0.03; 0.62)	0.26 (0.13; 0.45)	p_1_ = 0.531 p_2_ = 0.945 p_3_ = 0.728
Mature ‘naïve’ B cells (CD27−CD38+)	6.82 (3.05; 9.11)	7.51 (3.97; 11.73)	5.36 (2.84; 8.2)	p_1_ = 0.682 p_2_ = 0.664 p_3_ = 0.295
Mature activated B cells (CD27+CD38+)	2.18 (1.06; 3.26)	1.36 (1.01; 1.96)	1.4 (0.88; 2.57)	p_1_ = 0.068 p_2_ = 0.223 p_3_ = 0.81
Plasmablasts (CD27++CD38++)	0.07 (0.02; 0.1)	0.03 (0.02; 0.07)	0.03 (0.02; 0.04)	p_1_ = 0.374 p_2_ = 0.133 p_3_ = 0.979
Resting memory B cells (CD27+CD38−)	1.87 (0.79; 2.35)	0.76 (0.39; 1.27)	1.33 (0.82; 2.72)	p_1_ = 0.068 p_2_ = 0.664 p_3_ = 0.098
Double-negative B cells (CD27−CD38−)	0.72 (0.36; 1.16)	0.39 (0.23; 0.83)	0.77 (0.49; 1.42)	p_1_ = 0.232 p_2_ = 0.568 p_3_ = 0.168
‘naïve’ B cells (IgD+CD27−)	7.69 (3.74; 9.74)	9.55 (5.18; 12.97)	6.78 (3.67; 9.49)	p_1_ = 0.559 p_2_ = 0.802 p_3_ = 0.27
‘unswitched’ memory B cells (IgD+CD27+)	2.03 (0.68; 2.85)	0.98 (0.42; 1.58)	1.09 (0.79; 2.45)	p_1_ = 0.075 p_2_ = 0.423 p_3_ = 0.347
‘switched’ memory B cells (IgD−CD27+)	1.83 (1.05; 2.67)	1.01 (0.62; 1.31)	1.43 (0.74; 1.88)	p_1_ = 0.045 p_2_ = 0.174 p_3_ = 0.295
Double-negative B cells (IgD−CD27−)	0.49 (0.25; 0.77)	0.37 (0.28; 0.49)	0.32 (0.15; 0.47)	p_1_ = 0.475 p_2_ = 0.1 p_3_ = 0.611
Plasmablasts (IgD−CD27++)	0.02 (0.01; 0.05)	0.01 (0.01; 0.02)	0.02 (0.01; 0.02)	p_1_ = 0.249 p_2_ = 0.507 p_3_ = 0.611

p_1_—statistical differences between patients with CD and patients with AS associated with CD; p_2_—statistical differences between patients with CD and patients with AS; p_3_—statistical differences between patients with AS associated with CD and patients with AS. The relative number of B cells was calculated as a ratio to total lymphocytes. The differences between the groups were estimated using Mann–Whitney U-test without correction for multiple testing. The quantitative data are presented as median and quartile ranges (Med (Q25; Q75)).

## Data Availability

The original contributions presented in this study are included in the article/Supplementary Material. Further inquiries can be directed to the corresponding author.

## Supplementary Materials

The following supporting information can be downloaded at https://www.mdpi.com/article/10.3390/cells15171580/s1: Figure S1: Flow cytometry immunophenotyping gating strategy for B cells; Table S1: List of monoclonal antibodies for immunophenotyping of peripheral blood B-cell subsets; Figure S2: T cells’ flow cytometry immunophenotyping gating strategy; Table S2: List of monoclonal antibodies for immunophenotyping of peripheral blood T-cell subsets; Figure S3: Gating strategy for regulatory T cells; Table S3: List of monoclonal antibodies for immunophenotyping of peripheral blood regulatory T-cell subsets; Table S4: ‘Polarized’ Th subsets in patients with CD, AS+CD, and AS; Table S5: Treg maturation subsets in patients with CD, AS+CD, and AS.

## Abbreviations

The following abbreviations are used in this manuscript:

AS	Ankylosing spondylitis
SpA	Spondyloarthritis
GIT	Gastrointestinal tract
CD	Crohn’s disease
IBD	Inflammatory bowel disease
ASAS	Assessment of SpondyloArthritis international Society
axSpA	Axial Spondyloarthritis
CT	Computed tomography
SIJ	Sacroiliac joints
ILC	Innate Lymphoid Cell
HC	Healthy controls
BASDAI	Bath Ankylosing Spondylitis Disease Activity Index
ASDAS	Ankylosing Spondylitis Disease Activity Score
CDAI	Best’s Crohn’s Disease Activity Index
csDMARDs	Conventional synthetic disease-modifying antirheumatic drugs
bDMARDs	Biological disease-modifying antirheumatic drugs
NSAIDs	Non-steroidal anti-inflammatory drugs
SterRes	Corticosteroid resistance
SterDep	Corticosteroid dependency
RgSI	Radiographic sacroiliitis
MRSI	MRI sacroiliitis
RgSynd	Radiographic spondylitis
MRspond	MRI spondylitis
IBP	Inflammatory back pain
UlcEros	Intestinal ulcers and erosions
HBI	Harvey-Bradshaw Index
BASFI	Bath Ankylosing Spondylitis Functional Index
BASMI	Bath Ankylosing Spondylitis Metrology Index
BASMIten	Bath Ankylosing Spondylitis Metrology Index 10-point scale
MASES	Maastricht Ankylosing Spondylitis Enthesis Score
Treg	Regulatory T cell
Tcyt	Cytotoxic T cell
Th	T-helper cell
CM	Central memory
EM	Effector memory
TEMRA	Terminally differentiated CD45RA-positive effector memory cell
tSNE	t-Distributed Stochastic Neighbor Embedding
DN	Double-negative
DP	Double-positive
Tfh	T-follicular helper cell
CRP	C-reactive protein
ESR	Erythrocyte sedimentation rate
IL	Interleukin
TNF	Tumor Necrosis Factor Alpha
IFN	Interferon
pSS	Primary Sjögren’s syndrome
IgG4-RD	IgG4-Related Disease
CD-axSpA	Crohn’s disease-associated axial spondyloarthritis
SCCAI	Simple Clinical Colitis Activity Index
tsDMARD	Targeted synthetic disease-modifying anti-inflammatory drugs
