# Supplementary material for: Comprehensive Phenotyping of Circulating T- and B-Cell Subsets in Patients with Ankylosing Spondylitis Associated with Crohn’s Disease
Source: Cells. 2026 Aug 31;15(17):1580. doi: 10.3390/cells15171580 (PMC13565554; doi:10.3390/cells15171580)
Supplement: Supplementary file 1 [file cells-15-01580-s001.zip › cells-4485084-supplementary.pdf]

Figure S1. Flow cytometry immunophenotyping gating strategy for B cells.

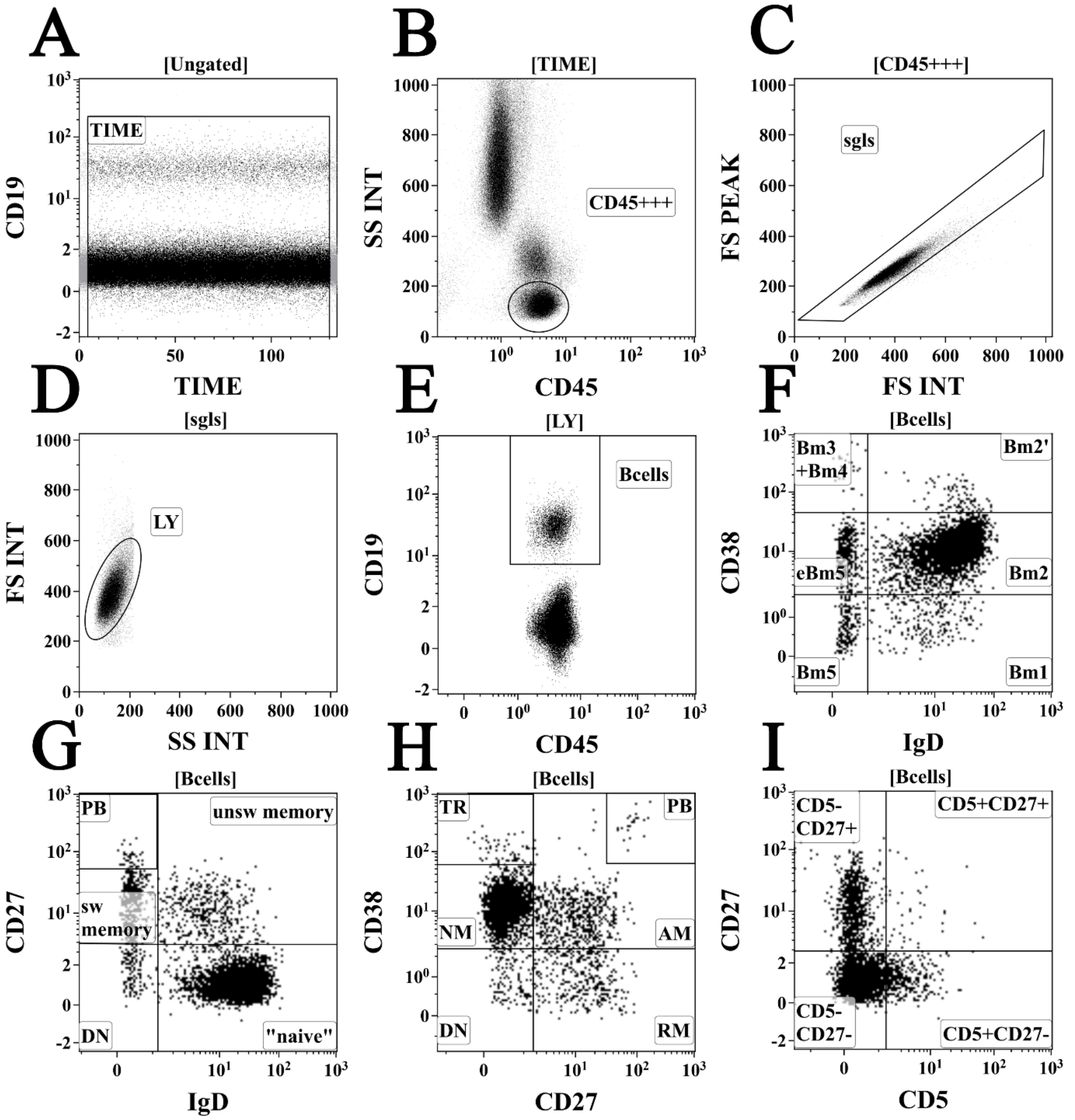

Description: **(A)** artifact exclusion included time gating; **(B)** total lymphocyte subset purification based on side scatter and bright CD45 expression; **(C)** doublets exclusion from the analysis by using the ratio between integral and peak forward scatter signals; **(D)** discrimination between lymphocytes and cell debris; **(E)** total CD19 expression-based B cell subset gaiting; **(F)** Co-expression of IgD and CD38 (so-called "Bm1-Bm5" classification) allowed to purify "virgin naïve" Bm1 cell IgD+CD38-, "activated naïve" Bm2 cells (IgD+CD38+), pre-germinal-center Bm2′ cells (IgD+CD38++), common subset, containing centroblasts and centrocytes (so-called "Bm3+Bm4" cells, IgD-CD38++), early memory and resting memory cells (eBm5 and Bm5 cells with the following phenotypes – IgD-CD38+ and IgD-CD38-, respectively); **(G)** Co-expression of IgD and CD27 allowed to purify "naïve" cells (IgD+CD27–) and three types of memory cells – "unswitched" memory cells ('unsw' IgD+CD27+), "class-switched" memory cells (memory IgD–CD27+) and co-called "double-negative" memory cells (DN IgD–CD27–); **(H)** Co-expression of CD27 and CD38 allowed to purify transitional B cells (CD27–CD38++), mature 'naïve' B cells (CD27–CD38+), mature activated B cells (CD27+CD38+), plasmablasts (CD27++CD38++), resting memory B cells (CD27+CD38–), and double-negative B cells (CD27–CD38–); **(I)** CD5-expressing B cell were purified within total B cell subset.

Table S1. List of monoclonal antibodies for immunophenotyping of peripheral blood B cell subsets.

N	Antigen	Fluorochrome	Clone	Isotype	Cat. number	Manufactured by
**1**	IgD	Alexa Fluor 488	IA6-2	Mouse IgG2a, κ	348216	BioLegend, Inc., USA
**2**	CD38	PE	LS198-4-3	Mouse IgG1, κ	A07779	Beckman Coulter, USA
**3**	CD27	PC7	1A4CD27	Mouse IgG1, κ	A54823	Beckman Coulter, USA
**4**	CD19	APC/Cy7	HIB19	Mouse IgG1, κ	302218	BioLegend, Inc., USA
**5**	CD5	Pacific Blue	BL1a	Mouse IgG2a, κ	A82790	Beckman Coulter, USA
**6**	CD45	Krome Orange	J33	Mouse IgG1, κ	A96416	Beckman Coulter, USA

Figure S2. T cells flow cytometry immunophenotyping gating strategy.

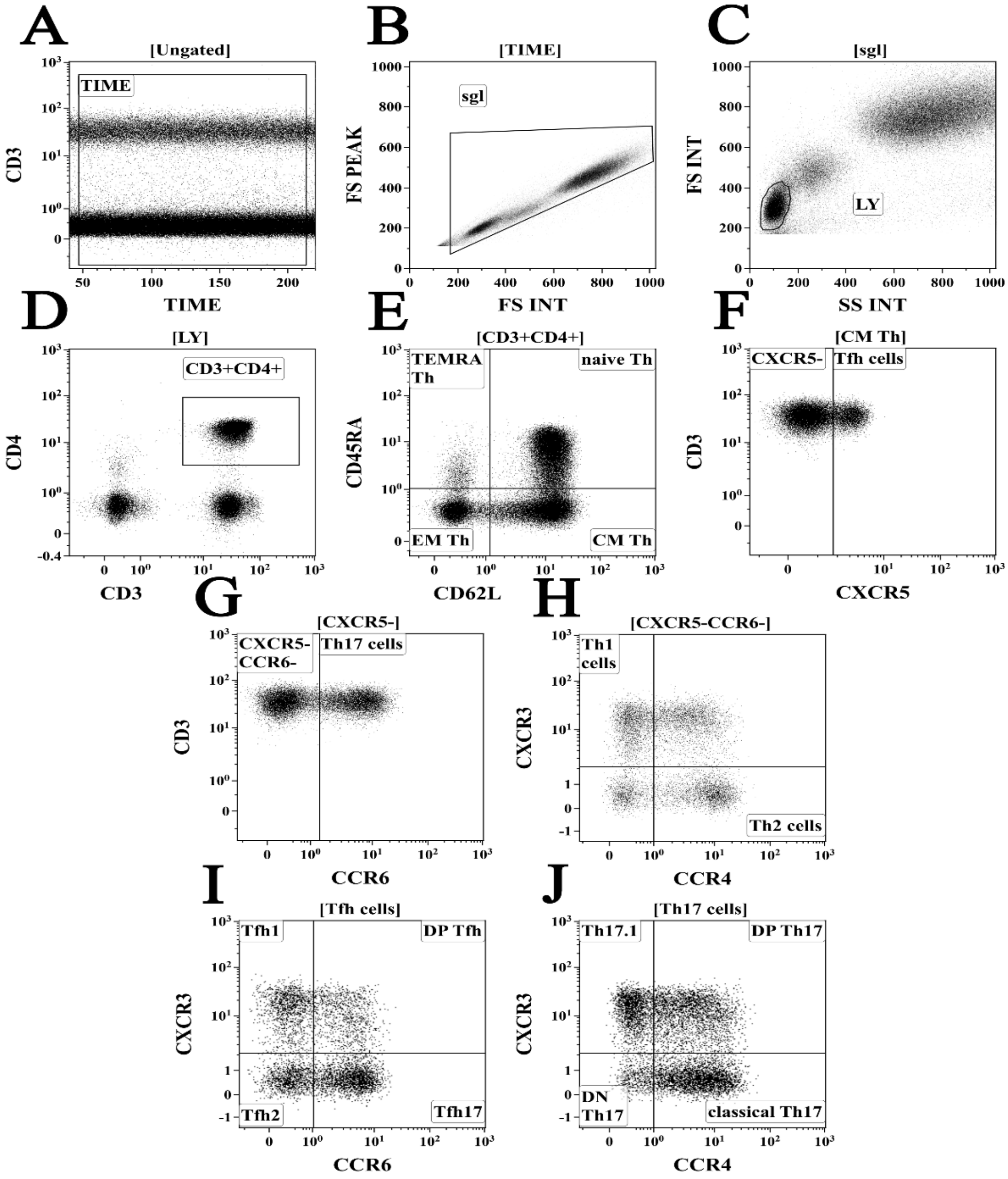

Description: **(A)** artifact exclusion included time gating; **(B)** doublets exclusion from the analysis by using the ratio between integral and peak forward scatter signals; **(C)** total lymphocyte population was gated based on their side-scatter and forward-scatter; **(D)** Th cells were further analyzed by expression of CD3 and CD4; **(E)** CD45RA and CD62L co-expression in identifying the four major CD4+ T cell maturation subsets: 'naïve' CD45RA+CD62L+, central memory CD45RA–CD62L+ (CM), effector memory CD45RA−CD62L− (EM), and terminally differentiated CD45RA-positive effector memory CD45RA+CD62L− (TEMRA) CD4+ T cells; **(F)** based on CXCR5 expression the total Tfh cell subset was identified; **(G)** expression of CCR6 by Th cells; **(H)** based on CXCR3 and CCR4 expression Th1 and Th2 subsets was identified; **(I)** determining Tfh cell-surface expression of the chemokine receptors CXCR3 and CCR6 allowed to identify CXCR3+CCR6− Tfh1-, CXCR3−CCR6− Tfh2-, CXCR3–CCR6+ Tfh17-like as well as Th17.1-like (double-positive) Tfh cells; **(J)** determining expression of the chemokine receptors CXCR3 and CCR4 on total Th17 surface allowed to identify 'classical' CCR6+CCR4+ Th17 cells, 'non-classical' CCR6+CXCR3+ Th17.1 cells, double-negative (DN) Th17 cells lacking both CXCR3 and CCR4, double-positive (DP) Th17 cells co-expressing CXCR3 and CCR4.

Table S2. List of monoclonal antibodies for immunophenotyping of peripheral blood T cell subsets.

N	Antigen	Fluorochrome	Clone	Isotype	Cat. number	Manufactured by
**1**	CD45RA	FITC	ALB11	Mouse IgG1, κ	IM0584U	Beckman Coulter, USA
**2**	CD62L	PE	DREG56	Mouse IgG1, κ	IM2214U	Beckman Coulter, USA
**3**	CXCR5 (CD185)	PerCP/Cy5.5	J252D4	Mouse IgG1, κ	356910	BioLegend, Inc., USA
**4**	CCR6 (CD196)	PE/Cy7	G034E3	Mouse IgG2b, κ	353418	BioLegend, Inc., USA
**5**	CXCR3 (CD183)	APC	G025H7	Mouse IgG1, κ	353708	BioLegend, Inc., USA
**6**	CD3	APC-Alexa Fluor 750	UCHT1	Mouse IgG1, κ	A94680	Beckman Coulter, USA
**7**	CD4	Pacific Blue	13B8.2	Mouse IgG1, κ	B49197	Beckman Coulter, USA
**8**	CCR4 (CD194)	Brilliant Violet 510	L291H4	Mouse IgG1, κ	359416	BioLegend, Inc., USA

Figure S3. Gating strategy for regulatory T cells.

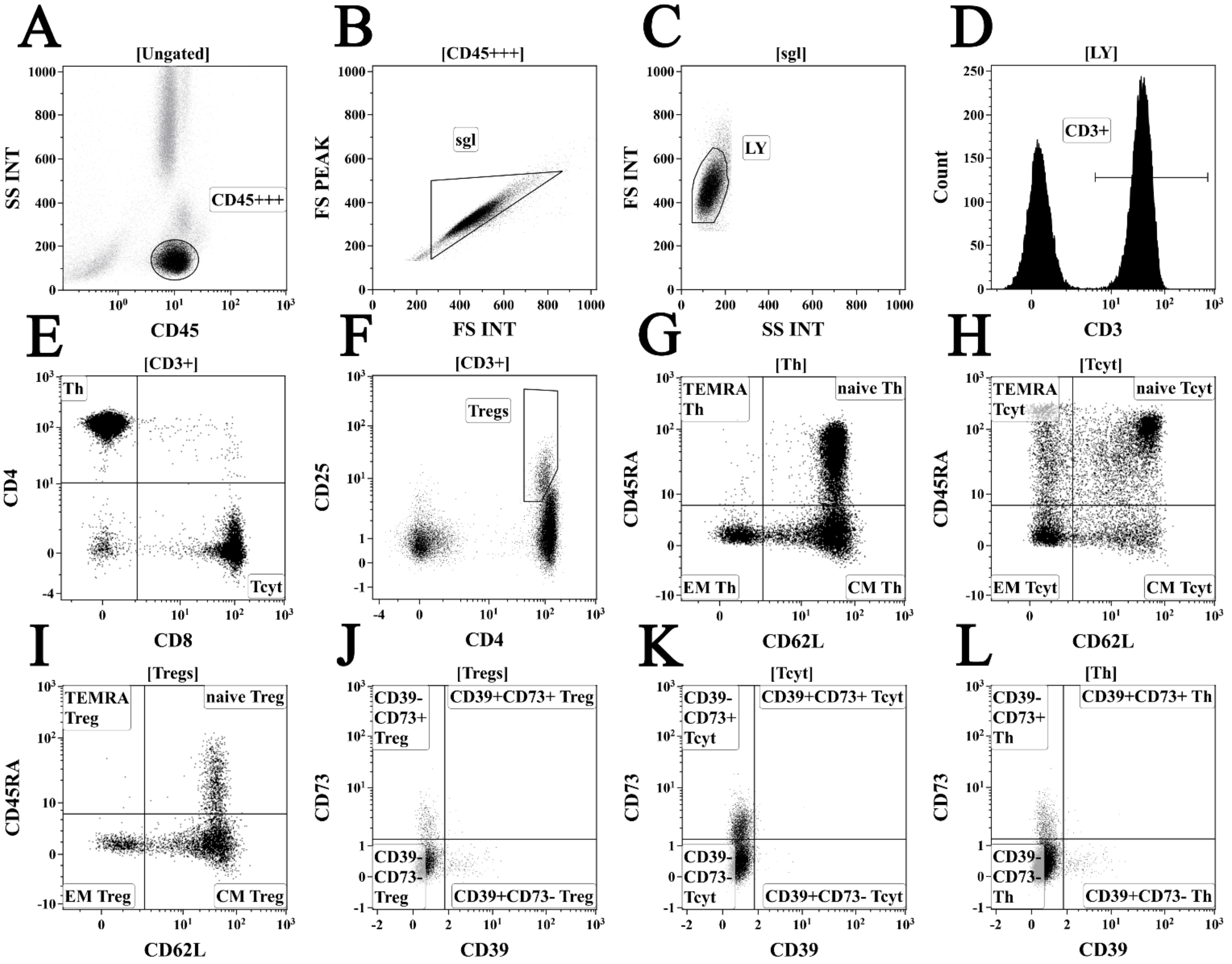

Description: **(A)** total lymphocyte subset purification based on side scatter and bright CD45 expression; **(B)** doublets exclusion from the analysis by using the ratio between integral and peak forward scatter signals; **(C)** total lymphocyte population was gated based on their side-scatter and forward-scatter; **(D)** total CD3 expression-based T cell subset gaiting; **(E)** CD4+ and CD8+ T cells detected within total CD3+ T cell population; **(F)** T cells were further analyzed by expression of CD25 and CD4 for identification of total regulatory T cells ("Tregs"); **(G)** CD45RA and CD62L co-expression in identifying the four major CD4+ T cell maturation subsets: 'naïve' CD45RA+CD62L+, central memory CD45RA–CD62L+ (CM), effector memory CD45RA−CD62L− (EM), and terminally differentiated CD45RA-positive effector memory CD45RA+CD62L− (TEMRA) CD4+ T cells; **(H)** CD45RA and CD62L co-expression in identifying the four major CD8+ T cell maturation subsets: 'naïve' CD45RA+CD62L+, central memory CD45RA–CD62L+ (CM), effector memory CD45RA−CD62L− (EM), and terminally differentiated CD45RA-positive effector memory CD45RA+CD62L− (TEMRA) CD8+ T cells; **(I)** CD45RA and CD62L co-expression in identifying the four major Tregs maturation subsets: 'naïve' CD45RA+CD62L+, central memory CD45RA–CD62L+ (CM), effector memory CD45RA−CD62L− (EM), and terminally differentiated CD45RA-positive effector memory CD45RA+CD62L− (TEMRA) CD8+ Tregs; **(J–L)** examples of CD39 and CD73 expression by Tregs, CD8+ T cells and CD4+ T cells, respectively.

Table S3. List of monoclonal antibodies for immunophenotyping of peripheral blood regulatory T cell subsets.

N	Antigen	Fluorochrome	Clone	Isotype	Cat. number	Manufactured by
**1**	CD39	FITC	A1	Mouse IgG1, κ	328206	BioLegend, Inc., USA
**2**	CD25	PE	B1.49.9	Mouse IgG2a, κ	A07774	Beckman Coulter, USA
**3**	CD62L	ECD	DREG56	Mouse IgG1, κ	IM2713U	Beckman Coulter, USA
**4**	CD45RA	PerCP/Cyanine5.5	HI100	Mouse IgG2b, κ	304122	BioLegend, Inc., USA
**5**	CD4	PC7	SFCI12T4D11 (T4)	Mouse IgG1, κ	737660	Beckman Coulter, USA
**6**	CD8	APC	B9.11	Mouse IgG1, κ	IM2469	Beckman Coulter, USA
**7**	CD3	APC-Alexa Fluor 750	UCHT1	Mouse IgG1, κ	A94680	Beckman Coulter, USA
**8**	CD73	Pacific Blue	AD2	Mouse IgG1, κ	344012	BioLegend, Inc., USA
**9**	CD45	Krome Orange	J33	Mouse IgG1, κ	A96416	Beckman Coulter, USA

Table S4. ‘Polarized’ Th subsets in patients with CD, AS+CD, and AS.

	CD (n = 16)	AS+CD (n = 13)	AS (n = 13)	Significant differences
Th1	12.36 (9.86; 18.89)	11.23 (7.71; 14.26)	10.34 (7.55; 12.19)	$p_1 = 0.398$<br>$p_2 = 0.075$<br>$p_3 = 0.39$
Th2	4.74 (2.52; 7.21)	5.19 (4.51; 6.46)	5.73 (5.37; 6.43)	$p_1 = 0.531$<br>$p_2 = 0.156$<br>$p_3 = 0.169$
total Th17	30.32 (17.19; 35.78)	23.22 (17.94; 26.72)	21.67 (19.39; 30.68)	$p_1 = 0.215$<br>$p_2 = 0.475$<br>$p_3 = 0.545$
‘classical’ Th17	31.12 (16.49; 40.32)	27.25 (24.93; 40.1)	38.38 (32.94; 45.91)	$p_1 = 1$<br>$p_2 = 0.092$<br>$p_3 = 0.05$
Th17.1	24.64 (19.55; 38.33)	34.75 (26.14; 40.56)	27.52 (21.44; 31.44)	$p_1 = 0.351$<br>$p_2 = 0.914$<br>$p_3 = 0.113$
DN Th17	10.62 (7.63; 15.86)	11.89 (8.34; 13.22)	14.11 (8.93; 16.52)	$p_1 = 0.948$<br>$p_2 = 0.449$<br>$p_3 = 0.418$
DP Th17	19.55 (17.29; 29.23)	22.94 (19.3; 30.39)	19.86 (17.46; 26.93)	$p_1 = 0.398$<br>$p_2 = 0.812$<br>$p_3 = 0.223$
Tfh	5.74 (4.28; 7.01)	5.49 (3.88; 7.67)	7.03 (4.27; 8.01)	$p_1 = 0.812$<br>$p_2 = 0.503$<br>$p_3 = 0.545$

p1—statistical differences between patients with CD and patients with AS associated with CD; p2—statistical differences between patients with CD and patients with AS; p3—statistical differences between patients with AS associated with CD and patients with AS. The statistical analysis was performed with Kruskal Wallis test with post-hoc Dunn’s test. The differences between the groups were estimated using Mann–Whitney U-test without correction for multiple testing. The quantitative data are presented as median and quartile ranges (Med (Q25; Q75).

Table S5. Treg maturation subsets in patients with CD, AS+CD, and AS.

	CD (n = 16)	AS+CD (n = 13)	AS (n = 13)	Significant differences
'naïve' Treg	24.34 (15.67; 42.59)	33.79 (22.04; 48.92)	24.96 (19.79; 33.66)	$p_1 = 0.351$<br>$p_2 = 0.909$<br>$p_3 = 0.186$
CM Treg	57.36 (44.68; 60.48)	43.13 (25.69; 58.38)	58.39 (46.56; 60.55)	$p_1 = 0.232$<br>$p_2 = 0.698$<br>$p_3 = 0.152$
EM Treg	16.14 (12.16; 22.77)	11.42 (10.04; 19.37)	14.04 (9.59; 19.11)	$p_1 = 0.184$<br>$p_2 = 0.397$<br>$p_3 = 0.936$
TEMRA Treg	0.96 (0.43; 4.98)	0.73 (0.37; 8.74)	0.71 (0.3; 3.27)	$p_1 = 0.559$<br>$p_2 = 0.507$<br>$p_3 = 0.47$

p1—statistical differences between patients with CD and patients with AS associated with CD; p2—statistical differences between patients with CD and patients with AS; p3—statistical differences between patients with AS associated with CD and patients with AS. The statistical analysis was performed with Kruskal Wallis test with post-hoc Dunn's test. The differences between the groups were estimated using Mann–Whitney U-test without correction for multiple testing. The quantitative data are presented as median and quartile ranges (Med (Q25; Q75).
